# Alcohol industry involvement in policymaking: a systematic review

**DOI:** 10.1111/add.14216

**Published:** 2018-05-06

**Authors:** Jim McCambridge, Melissa Mialon, Ben Hawkins

**Affiliations:** ^1^ Department of Health Sciences University of York York UK; ^2^ Faculty of Public Health and Policy London School of Hygiene and Tropical Medicine London UK

**Keywords:** Alcohol, alcohol industry, alcohol policy, corporate, framing, policy making, policy process

## Abstract

**Aims:**

To summarize the substantive findings of studies of alcohol industry involvement in national or supranational policymaking, and to produce a new synthesis of current evidence.

**Methods:**

This study examined peer‐reviewed journal reports published in the English language between 1980 and 2016 of studies of alcohol industry involvement in policymaking. Included studies were required to provide information on data collection and analysis and to have sought explicitly to investigate interventions by alcohol industry actors within the process of public policymaking. Eight electronic databases were searched on 27 February 2017. The methodological strengths and limitations of individual studies and the literature as a whole were examined. A thematic synthesis using an inductive approach to the generation of themes was guided by the research aims and objectives.

**Results:**

Twenty reports drawn from 15 documentary and interview studies identify the pervasive influence of alcohol industry actors in policymaking. This evidence synthesis indicates that industry actors seek to influence policy in two principal ways by: (1) framing policy debates in a cogent and internally consistent manner, which excludes from policy agendas issues that are contrary to commercial interests; and (2) adopting short‐ and long‐term approaches to managing threats to commercial interests within the policy arena by building relationships with key actors using a variety of different organizational forms. This review pools findings from existing studies on the range of observed impacts on national alcohol policy decision‐making throughout the world.

**Conclusions:**

Alcohol industry actors are highly strategic, rhetorically sophisticated and well organized in influencing national policymaking.

## Introduction

The policies regarded by the research community as most likely to be effective in reducing alcohol harms are those which regulate the behaviour of industry actors, such as controlling the availability and increasing prices of alcohol 1. These policy measures place significant constraints on business practices. By contrast, dedicated national alcohol policies frequently appear to reflect the preferences of alcohol industry actors 2, 3, 4. They tend to afford considerable scope for industry self‐regulation and promote non‐regulatory measures, eschewing effective population‐level policy measures which address the alcogenic environment created by the commercial activities of corporate actors 5.

Studies in other policy areas, such as tobacco control or environmental protection, have identified a range of corporate political activities designed to shape policy, including attempts, for example, to shape the evidentiary content of policy debates 6, 7, 8, 9. The research literature examining alcohol industry policy influence appears to have emerged quite recently in response to the articulation of serious concerns by the research community 3, 10. Knowledge of the alcohol industry's putative influence on policy could be strengthened with evidence from studies that investigate the involvement of industry actors in policymaking. Although the need to undertake this kind of research has been identified in the field of public health 4, 11, 12, studies of the political activities of alcohol industry actors may also exist in other disciplines. Apart from one systematic review concerned with marketing regulation 13, no other evidence synthesis is known to have been undertaken on alcohol industry actors' involvement in policymaking. The existing systematic review emphasized the similarities which exist with the tobacco industry in both the tactics used to influence policy and the framing of arguments promoted to shape policy debates 13. The present study focuses on the ways in which alcohol industry actors are involved in policymaking, and is not concerned with policy implementation or with the outcomes of policy decisions.

## Methods

This systematic review 14 examines only studies published in peer‐reviewed journals, as it is not judged possible to identify and appraise grey literature in an unbiased manner. For the purposes of this study, the alcohol industry is defined as economic actors involved in the production, distribution and marketing of alcohol 15 (regardless of whether this is the primary feature of their business), as well as trade associations and social aspects organizations 16. The aim of this systematic review is to investigate existing empirical evidence on alcohol industry actors' involvement in policymaking. The study objectives are as follows:

1. To summarize the substantive findings of existing studies;
2. To assess the strengths and the limitations of existing research;
3. To synthesize existing evidence across studies; and
4. To identify key gaps in current evidence.

To be included in this review, studies should:

- Be published in peer‐reviewed journals during the period 1980–2016 inclusive;
- Be published in the English language;
- Seek explicitly to study (for example, as reflected in stated aims) interventions by alcohol industry actors within the process of public policymaking;
- Provide information on data collection and analysis processes in a dedicated Methods section to enable an assessment of methodological strengths and limitations. For reviews to be eligible they must search more than one database, have explicit selection criteria and report on all studies included (if not they were treated as discussion papers and excluded);
- Present data on alcohol industry actors separately in studies of corporate activities across multiple sectors and/or of multiple actors within policy process; and
- Be concerned with policy at the national or supranational levels.

Commentaries, editorials, letters, discussion papers and conceptual work on alcohol industry actors are excluded, including one integrative review of a set of primary studies included here 17. Also excluded are studies of industry activities not concerned directly with influencing policy, such as in relation to evidence production, corporate social responsibility or the direct consequences of commercial activities, such as marketing, on public health. The earliest date is chosen to include data prior to the concentration of ownership of alcohol producers by a small number of global alcohol corporations which occurred since the 1990s 15. Data from studies of supranational policy levels (e.g. European Union) are understood to be informative about national level political activities. The subnational policy level is excluded, as it is anticipated that the nature of alcohol industry policy involvement may be importantly different, as suggested by an existing study at this level with a somewhat different focus 18.

Literature search strategies were developed using Medical Subjects Headings (MeSH) terms and key word terms. Previous systematic reviews in related areas were consulted in iteratively developing both the search terms and the databases to be included. Eight different health and social science databases were searched: Web of Science Core Collection; BIOSIS Citation Index; SciELO Citation Index; CINAHL Plus; Embase; MEDLINE; PsycINFO; and Scopus. The basic search strategy was organized around the three constructs of ‘alcohol’, ‘industry’ and ‘policy’ (corporat* OR industr* OR compan* OR busines* OR firm*) AND (alcohol OR drink) AND (marketing OR advertis* OR sponsor*) AND (regulat* OR policy OR legislat*) and developed with the support of a specialist librarian. The search strategy for MEDLINE is presented in Supporting information, Appendix S1. Searches were conducted on 27 February 2017. We did not register nor publish a protocol for this review.

The second author led data collection, identifying additional material in non‐database searching, including backwards and forwards citation searches, hand‐searching the special series on policy case studies and vested interests within this journal and contacting topic experts. Titles and abstracts were downloaded and imported to EndNote (where duplicates were removed). Titles (and abstracts if available) were screened, with 10% of all records checked by the first author. Potentially eligible full texts were obtained, and eligibility of all material was determined by the first two authors by assessing papers independently against the selection criteria in Excel, then discussing any disagreements.

A thematic analysis using an inductive approach to the generation of themes 19, 20 was guided by the review aims and objectives, mindful that this was an apples‐and‐oranges review of studies with diverse study designs and foci. This involved progression from stages of coding and summarizing thematic material within the included studies to a final stage of going beyond the themes as they were reported 21. In approaching the analysis in this way, we thus decided at the outset to eschew the adoption of a pre‐existing conceptual framework such as the one based on the tobacco industry used by Savell and colleagues 13. This approach was intended to produce a novel investigation of the extent of similarities with the tobacco industry and other themes that was complementary to that used by Savell and colleagues 13. In keeping with the handling of the substantive findings, and in preference to formal assessment of risk of bias, the methodological examination avoided the application of in‐depth analytical techniques, with details being provided on data collection and analysis alongside observations on study limitations.

We began by reading all included reports, with the third author leading the development and refinement of the initial thematic coding and extraction of material using direct capture of relevant text via cut‐and‐paste into tables in Microsoft Word. This was accompanied by a precis of key findings within each analytical category for all included reports (see Supporting information, Appendix S2 for this data set). In the next stage, the analysis moved from examination of relevant content within studies to the aggregation of themes across studies. The thematic summaries of data from primary studies are presented parsimoniously in Tables 2, 3, 4. The first and third authors worked closely together throughout this process. Following brief methodological characterization of the literature, the narrative presentation in the Results section is concerned primarily with generating a novel synthesis.

## Results

**Figure 1:**
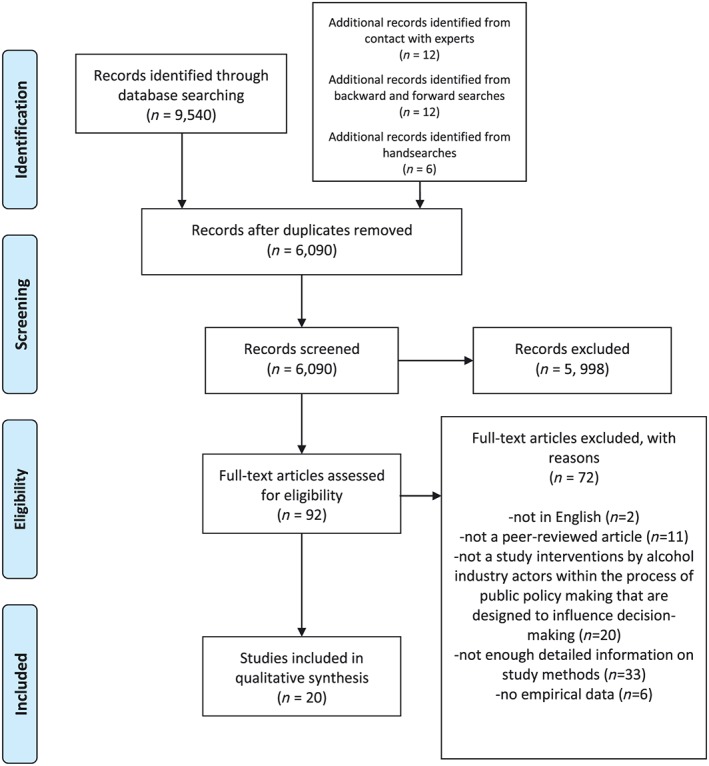
Preferred Reporting Items for Systematic Reviews and Meta‐Analyses (PRISMA) flow‐chart [Colour figure can be viewed at http://wileyonlinelibrary.com]

This review identified 20 reports drawn from 15 studies (treating each study as a discrete data collection exercise, with some generating multiple journal reports) as eligible for inclusion [see Figure 1]. These were largely published since 2012 (six reports 22, 23, 24, 25, 26, 27 from four studies were earlier) and concerned mainly high‐income, English‐speaking countries [seven UK (four studies), two US (one study), two Australia, one New Zealand], with other reports of studies undertaken in Africa (Lesotho, Malawi, Uganda and Botswana 26), Hong Kong 28, Thailand 29 and Poland 30, respectively. All are qualitative studies, with the exception of the Thai study 29, which largely presents quantitative data. There were three reports (two studies) based on internal tobacco company documents 24, 25, 31 and one systematic review on influence of marketing regulation without a particular geographical focus 13. These reports were all published in addictions or public health journals, apart from three in policy and politics journals 32, 33, 34.

Table 1 provides a summary of the characteristics of the included studies. Nine are solely documentary studies, four are case studies involving the presentation of a combination of documentary and other data sources and two are primarily interview studies, although both draw extensively on other data sources in the course of analyses.

**Table 1:** Characteristics of included studies.

	Study aims and review‐level observations on strengths of findings	Review‐level summary of data collection and analysis details	Review‐level observations on study limitations
Greenfield *et al*. 2004 23; Giesbrecht *et al*. 2004 22, a	This interview study draws on documentary data to investigate the US policy process and the handling of specific policy issues. There are important findings on industry arguments, political organization and influencing tactics (e.g. financial donations and lobbying) subsequently replicated, and also key data on policy processes; for example, the dynamics of the US federal policy system, that have not yet been studied further	Uses a rigorous two‐stage sampling approach to recruit multiple actor types involved in policymaking and obtains an impressive response rate in a large sample (*n* = 64). Detailed theoretically based, analytical procedures are described. These are linked to the analytical categories and validity issues are explicitly considered. Team analysis	Few details of how interviews were conducted in included reports. The difficulty of getting industry respondents to talk about policy dynamics is noted. Use made of documentary/archival data in the analysis somewhat unclear in included reports
Bond *et al*. 2009 24; Bond *et al*. 2010 25	This study of internal tobacco company documents takes advantage of Phillip Morris ownership of Miller Brewing Company to provide ‘smoking gun’ data on a range of key issues. It clearly identifies common policy concerns across tobacco and alcohol sectors. Policy issues are identified as key business risks globally, leading to an intention ‘to fight aggressively, with all available resources’ by ‘working behind the scenes’ through ‘a joint defence strategy’ with other alcohol industry actors and external lobbyists	Uses searching guidance for tobacco documents, identifying first 22 then increasing to 29 documents in the later paper on alcohol industry issues. Data unquestionably very strong. Thematic and content analyses are undertaken, although few details provided	Difficult to assess whether there may be other documents that have been missed. It may be possible to question external (i.e. generalizability to other actors) rather than internal validity. Later inclusion and coverage of Reynolds is somewhat unclear
Bakke & Endal 2010 26	This study uses documentary data along with external data in the manner of a case study. Resulted from the chance discovery of software confirming industry authorship of draft national alcohol policy documents in four African countries. Safer drinking for the entire population was emphasized despite 3/4 being non‐drinkers, and industry‐preferred policies proposed. The industry actor also sought to institutionalize their participation in the processes of monitoring and reviewing resulting policies. This is another ‘smoking gun’ study	Detailed textual comparisons of policy documents used. No methodological details provided for workshop and other data.	It is unclear what impacts industry involvement in drafting these documents had on the subsequent final policies, or if conduct of this study impacted on policy decision‐making. The nature of the unpublished observational, interview and e‐mail correspondence data used, and their analysis, are also unclear
Miller *et al*. 2011 27	This is a documentary study of alcohol industry actor submissions to an Australian public consultation on prevention and health and the extent to which they promoted Drinkwise, a social aspects organization, to demonstrate corporate social responsibility. Promotion of Drinkwise in all industry submissions is identified	The data set comprises nine industry submissions of a total of 33 relevant to alcohol. Thematic analyses were undertaken. The data set is modest and the reported findings stick appropriately close to it	There are few details provided of the conduct of the analyses. The study focus is somewhat narrow and the resulting data set small
Yoon & Lam 2012 28	This documentary study examines the policy debate over a zero beer and wine tax in Hong Kong. During an 8‐year period, industry actors came to coherently organize lobbying efforts to connect with key politicians. The importance of ideas in the evolution of the debates and the weak nature of public health advocacy in so doing are also emphasized	Alcohol and related industry material, media reports and government data sources are examined; 97 documents were analysed thematically. First author conducted the analyses	Examples of key search terms only are given, and similarly, indicative types of documents provided. It is thus unclear whether informative documents that would change study findings may have been missed. Few details of analytical methods are provided
Holden *et al*. 2012 35; Holden & Hawkins 2012 32; Hawkins & Holden 2013 34; Hawkins & Holden 2014 33	This interview study is reported in a series of papers which collectively offer an extensive and coherent account of industry wide organization, policy framing, political strategy and engagement with policy makers in relation to alcohol pricing in the UK during a period of policy controversy. Comparative analysis of Scotland and England was incorporated into the study design	Stakeholder analysis to identify interviewees via purposive and subsequent snowball sampling. High level of access to industry actors. Triangulation between interview respondents (industry and non‐industry actors) as well as with documents/external data sources. Two authors involved in theoretically based analyses. Data saturation considered	Low levels of participation by governmental actors. Possible limitations in novel use of interview alongside documentary data for undertaking framing analyses. Variability in depth of insights across objects of study
Jiang & Ling 2013 31	This study of internal tobacco company documents aimed to study alliances between tobacco and alcohol industries in the US in 1980s and 1990s. It specifies three main policy areas of collaboration (tax, air pollution and advertising), the industry actors involved and the organizational vehicles created or used for this purpose. It identifies the importance of co‐ownership in the identification of common interests, strategies and coalition building	Examples of the search terms used are given. Extensive use is made of memos in analysing the documents, although it is not clear how documents have been selected. The numbers of documents examined are reported by issue rather than overall	It is unclear which actors have been investigated and thus what may have been missed. The scope of this study, including examination of effects, is more focused on tobacco control than alcohol policy
McCambridge *et al*. 2013 38	This documentary study examines the use of evidence in alcohol industry actor submissions to a Scottish public consultation on population‐level policy measures that were strongly opposed by industry actors. Misrepresentations of the international scientific evidence on alcohol policies are identified along with other tactics in evidence use	The study uses extensive direct quotation and provides access to 27 submission documents by industry actors. Analyses involved comparisons with the international scientific evidence base and gave weight to the frequency and prominence of evidential claims	The data set is large and it is not clear whether additional findings may have been missed. There is limited detail provided of the conduct of the analyses
Hawkins & McCambridge 2014 37	This case study examined how a global producer funded a respected think tank to produce reports at crucial stages in the development of the UK government's alcohol strategy. The tactics used to promote industry interests were similar to those used by transnational tobacco corporations, particularly using other, apparently independent, actors to articulate industry arguments	A range of data sources and data collection activities are described including those which capture political events. The design and composition of the case study provides some conceptual framework for the data analysis	Other data sources may have enhanced the findings of the case study. The reports were heavily promoted and a key policy decision was controversially reversed, although the study is not able to directly link the two. The industry actor studied is part‐owned by a tobacco company and may be untypical of other actors
Katikireddi *et al*. 2014 36, a	This case study investigated the development of pricing policy in Scotland and identifies two contrasting framings of the nature of the alcohol‐related problems to be addressed, which dominate the underlying policy debate: ‘social disorder’ (promoted by some but not all industry actors) versus ‘health’. This study thus emphasizes the importance of framing in policy debates	Combines both interview and documentary data including textual and oral evidence to a parliamentary committee. Offers a sophisticated, theoretically based approach to data analysis with details provided of the process	The study provides key data on industry actors although the scope is broader, so that there are limitations in the reporting of industry actor specific findings. Nonetheless, the study identifies a key difference in framing between industry actors supporting and opposing the policy measure
Kypri *et al*. 2014 39	This is a documentary study of submissions to a New Zealand parliamentary committee on a proposed bill to increase the minimum purchase age for alcohol. Industry submissions were highly unified in their opposition to the bill in comparison to those from other sources. Industry actors sought to increase the numbers of submissions opposing the bill	A large data set of 178 submissions, mainly from the public, industry and NGOs was included. Template/thematic analysis was used with doubled coding of data and detailed presentation of findings based on coding	The bulk of the alcohol industry data is presented quantitatively rather than qualitatively. The scope of this study is concerned largely with comparisons between submissions from different types of actors rather than on the industry *per se*
Sornpaisarn & Kaewmungkun 2014 29	This case study examines taxation by beverage category in Thailand during a 20‐year period and efforts by the three dominant alcohol companies to influence decision‐making in line with their interests. The tax regime favoured the largest company and the other two used donations and access to prominent politicians, including the Prime Minister, to lobby for policy change. Conflicts between sectoral interests are emphasized	Multiple data sources used include quantitative analyses of taxation data, participant observations, media reports and parliamentary documents. Two specific events in the policy process are described. Content analyses performed with triangulation between data sources is described	There are few qualitative data originating from the content analyses presented, and it is not clear how data from different sources were used in reaching conclusions. The quantitative data do not include imports. It is not clear how informative events, other than the two described, may be
Avery *et al*. 2016 40	This documentary study of alcohol industry submissions to an Australian parliamentary inquiry into fetal alcohol spectrum disorders sought to investigate how industry actors contribute to policy development. As in other studies, it found promotion of vested interests including advocacy of ineffective policies, problem minimization and attacks on opponents	This study examines a small data set of five submissions made by four national and one state‐level (Western Australia) trade association covering the main sectors of alcohol production. Thematic analytical methods are used, although not described in detail	There appears little depth to the data available from industry actors. Generalizability to other issues, actors and cultures could be limited, although the findings are similar to those of other studies
Zatonski *et al*. 2016 30	This documentary study examined framing of the policy debate around an increase in spirits tax in Poland. Industry actors successfully promoted an economic framing of the policy debate in opposition to a health frame, especially in newspapers	This study adds to the literature on the importance of framing, and discursive strategies more broadly, in alcohol policy debates. Identifies precisely all dates and data sources (print media, spirits industry websites, governmental records and parliamentary debates) and 155 documents on the spirits tax were included. Theoretically informed analyses of framing, content analysis and thematic analysis, with origins and outcomes of codes presented. First author conducted analyses	Does not present search terms or strategy. Included data somewhat lacking in depth. Limited coverage of included data sources, e.g. no online media
Savell *et al*. 2016 13	This study sought to review alcohol industry attempts systematically to influence alcohol policies on marketing. Key arguments synthesized among 17 reports emphasized industry self‐regulation and individual drinker responsibility. Strong commonalities between tobacco and alcohol industry political activities were identified, with variations due most probably to policy context. This first evidence synthesis provides a major milestone in the development of the literature on alcohol industry policy influencing strategies	The 17 reports involve different forms of evidence and only two of 17 reports are included within the present study due to differing foci and study designs (for example, grey literature is excluded here). A rigorous narrative synthesis of included reports is described, with findings also reported in detail. This included involvement of all three authors in analysis, and double coding of all data. A careful comparison of findings with those from a parallel review on the tobacco industry was also undertaken	The report is not reported according to PRISMA guidelines and required content is lacking. Potentially eligible reports may have been missed. The evidential strengths and limitations of individual reports, and the literature as a whole, have not been discussed explicitly

a Other reports from these studies did not fulfil eligibility criteria for this review for lack of sufficient industry actor study focus or data. NGO = non‐governmental; PRISMA = Preferred Reporting Items for Systematic Reviews and Meta‐Analyses.

Among the six non‐documentary studies, three (the UK studies by Holden and colleagues 32, 33, 34, 35 and Katikireddi and colleagues 36 and the US study by Giesbrecht and colleagues 22, 23) are designed carefully with detailed and rigorous attention to inference generation and reported transparently in studies generating multiple reports in the peer‐reviewed literature (see Table 1). These methodological strengths produce findings in which there can be a high degree of confidence. The other three 26, 29, 37 are more opportunistic in nature, with study conduct motivated by particular events yielding individual reports; one being a ‘smoking gun’ study, revealing industry actor authorship of draft national policy documents 26.

Four 27, 38, 39, 40 of the nine documentary studies examine public consultation submission data, two analyse broader policy‐related data sources 28, 30, two draw upon tobacco company documents 24, 25, 31 and one reviews existing research literature 13. There are limitations to the nature and extent of the data available for study in all four studies 27, 38, 39, 40 of public consultation data (see Table 1). The most rigorously conducted and reported consultation submission study by Kypri and colleagues 39 has a limited focus on industry actors.

### Framing arguments

As can be seen in Table 2, framing has been studied extensively, and this synthesis identifies three main strands, not only to the content but also to the strategic use of arguments made by alcohol industry actors within policymaking. Industry actors give a great deal of attention to how they themselves are regarded; they position themselves as vital stakeholders in policy debates and key partners to government in policy formulation and implementation. This positioning legitimates their interventions in policy debates, including their attempts to define the scale and nature of alcohol problems that policy measures should strive to address (see Table 2).

**Table 2:** Policy‐framing strategies.

Object of framing	Strategy
Policy actors	Position themselves as key stakeholders in the policymaking process and partners in tackling alcohol harms 13, 26, 33, 37, 38
	Position themselves as key economic actors; i.e. generators of tax revenue/employment 13, 23, 28, 30, 32, 34
	Claim they are responsible actors, unfairly demonized by public health actors and policymakers 13, 22, 30, 31
	• Emphasize that they are a legal industry 13, 26, 27, 34
	• Differentiate themselves from the tobacco industry 13
	Present public health actors as extremists (or neo‐prohibitionists) driven by a moral agenda in order to undermine their credibility and policy influence 23, 24, 25, 40
The policy problem	Play down the scale of alcohol problems (and thus the need for policy interventions) 13, 22, 24, 26, 27, 28, 30, 32, 34, 36, 37, 38, 40
	• frame the alcohol problem in terms of a small minority of problem drinkers versus the moderate majority who are already aware of the need to drink responsibly 13, 22, 24, 27, 32, 34, 36, 38, 40
	• emphasize positive effects of alcohol; e.g. social and health benefits of ‘moderate’ drinking 13, 26, 28, 30, 34
	• focus policy debates on narrow range of harms, issues and subpopulations; i.e. binge and youth drinking; drink driving; drinking in pregnancy; certain areas of the country 24, 26, 32, 34, 36, 37
	Promote individualized accounts of the nature of alcohol problems 13, 23, 24, 26, 27, 28, 32, 34, 36, 37, 38, 39
	• consumer behaviour (misuse), not the product, is the source of harm 13, 24, 34, 39
	• it is unfair to penalize the majority for the actions of the few 24, 27, 28, 34, 38
	Present alcohol and ‘responsible’ drinking as socially acceptable, while alcohol misuse should be socially unacceptable 22, 24, 26, 34, 36, 38
Policy positions	Oppose the whole population approach and specific measures derived from it; argue they are ineffective ‘blunt instruments’ which fail to address the real policy problems and have unintended negative consequences 13, 27, 34, 36, 38, 40
	Oppose:
	• minimum unit pricing (UK); argued it is ineffective, illegal and counterproductive; and that it unfairly targets moderate and less wealthy drinkers 32, 34, 36, 37, 38, 39
	• tax increases (except as a ‘less bad’ alternative to MUP) 24, 25, 28, 29, 30, 31, 32, 34
	• advertising, marketing and sponsorship restrictions 13, 22, 24, 27, 31
	• mandatory product labelling regimes 24, 25, 40
	• reductions in blood alcohol levels in drink‐driving laws 24
	• increases in minimum purchase age 39
	Promote targeted interventions (as direct alternatives to whole population intervention), e.g. on parenting style 13, 23, 24, 26, 27, 32, 34, 36, 37, 38, 39
	Promote voluntary, co‐ and self‐regulatory initiatives and partnerships (as direct alternatives to mandatory regimes) 13, 22, 24, 25, 26, 27, 34, 40
	• for public information and education including product labelling 13, 22, 24, 26, 27, 34, 40
	• for advertising and marketing codes 13, 22
	Promote better enforcement of existing laws (i.e. underage sales and drink driving) as opposed to passing new laws 13, 24, 26, 34, 38, 40
	Promote the ideal of evidence‐based policy, but use evidence selectively to support their policy preferences 13, 22, 24, 26, 27, 28, 34, 36, 37, 38, 39

MUP = minimum unit pricing.

Positions on alcohol harms are articulated carefully to subtly endorse societal and public health concerns, and at the same time play down the scale of the problems. Industry actors attempt to shift understandings of harms, and the appropriate policy responses, from a population‐level understanding to one which places responsibility on individual consumers (and thus away from alcohol itself and industry commercial practices). This individual‐level framing of the causes of alcohol harms lends itself to a policy focus on a minority of drinkers. This depends on representations of ‘normal’ drinking from which problematic behaviour is differentiated (see Table 2).

The specific policy measures advocated by industry actors follow directly from these rhetorical foundations. Policy approaches which are concerned with addressing the drinking of the entire population are judged to be fundamentally misdirected, including policy measures that restrict the ability of industry actors to brand, advertise, sell and price their products. Preferable policies centre on partnership approaches with industry actors to education and information provision, and target putative causes of problems among particular subpopulations (see Table 2).

Rhetorical techniques include false dichotomies between opposed policies and industry favoured policies, as pursuing the former does not preclude governments from also pursuing the latter. Underpinning the industry framing of policy positions identified here are misleading claims about the effectiveness, and unintended consequences, of whole population measures and their supporting evidence‐base, and about weaker data which are used to support preferred approaches. This does not prevent industry actors making strong rhetorical commitments to evidence‐based policy. This synthesis identifies that all three objects of framing (actors, the policy problem, policy positions) are interconnected logically and strategically within an internally consistent, and mutually reinforcing, framing of the policy issues. Industry actors can make intuitively plausible, and highly nuanced, arguments that can appear compelling if they are allowed to go unchallenged.

### Influencing activities

Table 3 summarizes the available evidence in the research literature on the activities of industry actors within policymaking. Studies demonstrate that alcohol industry actors seek to be involved in all stages of the policymaking process, including public consultations, parliamentary committees and working groups, and with all relevant policy actors including government ministers and political advisers, civil servants, officials and technical advisers, members of parliaments and other political representatives, as do other corporate actors 41. Evidence shows that they are highly effective in policymaking process. The consistency of findings across the studies identified in Table 3 exists despite the studies examining different policy debates at different times and in different policymaking contexts.

**Table 3:** Policy‐influencing strategies.

	Strategy
Adopt multiple organizational forms	Proceed unilaterally: individual companies lobby government and other policy actors directly; limited mainly to large companies with sufficient access and resources 13, 26, 32, 33, 35, 36, 38, 39
	Pursue traditional forms of collective action: form, and participate in, trade associations and develop other forms of collective interest representation 13, 22, 23, 24, 30, 32, 33, 35, 36, 38, 39, 40
	Create novel forms of collective action: use social aspects organizations and ad‐hoc campaign groups to give the impression of independence and as additional channels of influence to 13, 27, 33, 37, 38
	• speak publicly and to government with industry framing
	• disseminate research and produce reports to promote industry‐favourable messages
	• deliver public information campaigns
	• implement self‐regulatory regimes (e.g. Portman Group on advertising)
	Use external agencies 33, 37
	• media and public relations consultancies used to shape terms of policy debates
	• consultancies used to produce reports on policy issues
	• funding think‐tanks creates the perception of independent authority
Engaging policy actors	Long‐term relationship building with key decision‐makers via regular formal and informal contacts including creating reciprocal obligations 13, 23, 26, 28, 32, 33, 40
	• financial contributions to political parties/campaigns 13, 23
	• personal contacts and informal networks between industry actors and other policy actors 23, 33
	• providing government with ‘policy goods’; e.g. information, expertise, and policy delivery via self‐regulatory regimes (individually and via trade associations and social aspects organizations) 13, 22, 33, 40
	Short‐term issue specific campaigns in response to events 13, 23, 28, 29, 30, 31, 32, 33, 36, 37
	• adapt strategies pragmatically to the policy context 23, 32, 33
	• venue shifting (e.g. Edinburgh to Westminster) 32, 33
	• legal challenges 13, 32
	Fund or disseminate policy relevant research with supportive findings to create a separate, circumscribed and self‐referential literature using think‐tanks, academics, consultancies and similar policy actors 13, 27, 29, 37, 38
	Constituency building with influential policy actors 22, 23, 24, 25, 28, 31, 32, 33

This synthesis identifies a key distinction can be made between what can be termed proactive or long‐term and reactive or short‐term influencing activities. Long‐term lobbying is designed to shape the wider policy environment, including the background assumptions about the nature and functions of policy, the roles of industry within the policy process and the terms in which policy debates are conducted. The maintenance and reinforcement of the policy framings described above is central to long‐term industry strategy. In addition, long‐term lobbying involves sustained efforts to build intimate, ongoing relationships with key policy actors and decision‐makers through frequent contacts and other forms of engagement. This normalizes the involvement of industry actors in policy processes, helps keep unfavoured issues off policy agendas, mainly consolidating a favourable *status quo*, as well as providing a basis for reactive lobbying in response to specific policy debates or initiatives that arise (see Table 3). Such reactive lobbying also offers opportunities to advance key discourses, although this is issue‐specific and designed to achieve specific policy objectives, often the avoidance of unfavourable forms of regulation. Short‐term lobbying on particular issues at particular times has been the focus of much of the existing research. The key findings of this synthesis are thus to highlight the role of long‐term as well as short‐term approaches, the relationship of the former to framing and the symbiosis between these elements in overall industry strategy.

This synthesis indicates that industry actors create and adapt different organizational forms to undertake these policy‐influencing activities, ranging from long‐standing forms of interest‐group representation whose histories span decades (e.g. trade associations) to ad‐hoc types of collaboration on specific issues, as required. Collaborations between industry actors recognize the benefits of negotiated, strong and unified positions on key issues. Industry actors, however, are highly pragmatic and adopt varied organizational forms and leadership arrangements throughout policy issues, between different countries and at different times.

The evolution of social aspects organizations is particularly noteworthy, given the claims they permit about independence from the industry in the context of the global concentration of production and also because their public relations and framing functions extend beyond the immediate contexts of policymaking. Individual companies make their own decisions about their political strategies, with larger companies having more options to consider, including acting alone. Third parties (i.e. from outside the alcohol industry) are also used to engage policy actors (see Table 3). Evidence suggests that adroit management of relationships between alcohol industry actors, and with other actors and industries, extends to great care being taken in rhetorical distancing from the tobacco industry, while collaborating carefully in some policy influencing activities, particularly where there is shared ownership of companies.

### Effects on policy outcomes

Table 4 summarizes data from existing studies on the range of observed impacts on policy decision‐making. The effects of both framing and long‐term lobbying are complex to study. The timing of any such evaluation study matters because, throughout countries, there are examples of persistence in industry activities over time being able to secure desired policy outcomes, even after early setbacks. The relative resource disadvantage of public health actors is implicated in this pattern (see Table 4). It would be misguided, therefore, to examine policy impacts in crude success/failure terms.

**Table 4:** Effects on policy outcomes.

Study	Availability of data and content of findings
Giesbrecht *et al*. 2004 22; Greenfield *et al*. 2004 23	Examined a wide range of issues in US alcohol policy throughout the 1980s and 1990s, with most detailed data available on the effects on advertising and product labelling policy outcomes. Initial success by public health actors to enact legislation on health warning labels on alcohol products was gradually countered by industry efforts to include labelling content promoting the health benefits of alcohol. Broadcast and alcohol industry groups ran TV public‐awareness campaigns as voluntary alternatives to legislation as part of a package that led to proposals for mandatory regulations being withdrawn. A voluntary ban on spirits advertising was maintained, again avoiding legislative restrictions. The opposition of public health and industry actors make alcohol policy development widely recognized as challenging, with the consequence of inhibiting the development of new policies, apart from in windows of opportunity
Bond *et al*. 2009 24; Bond *et al*. 2010 25	No data as not part of study design
Bakke & Endal 2010 26	An alcohol company was identified to be responsible for the creation of the original drafts of national alcohol policy documents in four African countries. The contents of the final policies in these countries were not studied to provide direct evidence of impacts on policy decision‐making
Miller *et al*. 2011 27	No data as not part of study design
Yoon & Lam 2012 28	Despite strong support for economic liberalism and vigorous lobbying by alcohol industry actors, the Hong Kong government was initially resistant to calls to reduce alcohol tax as this was seen as an important source of revenue. Gradually, during the 8‐year period of study, continued industry lobbying, in the face of relatively weak public health advocacy, led to a change in policy with Hong Kong going from a relatively high tax rate to zero duties on wine and beer
Holden *et al*. 2012 35; Holden & Hawkins 2012 32; Hawkins & Holden 2013 34; Hawkins & Holden 2014 33	Extensive access to policymaking in the UK Government system, supported by framing activities to promote themselves as partners in alcohol policy, has led to a situation in which industry actors are widely accepted as legitimate actors in the policy process, well positioned to obtain favourable policy outcomes. Following devolution within the UK, the election of a new party of government disrupted the equilibrium previously pertaining to alcohol policy in Scotland. This led to legislation on alcohol minimum unit pricing, despite extensive industry opposition. Public health actors were better able to gain access to policymaking in Scotland than they had been at the UK level, where the privileged access possessed by industry actors was maintained
Jiang & Ling 2013 31	Coalitions of tobacco and alcohol industry actors, particularly where shaped by instances of shared ownership, have had a number of successes in influencing tobacco control policy in the US. No data are provided on alcohol policies.
McCambridge *et al*. 2013 38	The alcohol industry submissions to a public consultation on alcohol policy examined in this study constituted one strand of industry strategies to influence alcohol policy in Scotland that were ultimately unsuccessful, as described elsewhere in this table
Hawkins & McCambridge 2014 37	This study examined the contribution of a global alcohol producer's partnership with a think tank to the reversal of a policy decision on alcohol minimum unit pricing in England. While not designed to draw strong conclusions, the study provides circumstantial evidence of industry influence on decision‐making.
Katikireddi *et al*. 2014 36	Framing by public health actors in support of alcohol minimum unit pricing was more successful than that used by industry actors in opposing the policy in Scotland (also see above)
Kypri *et al*. 2014 39	No data as not part of study design
Sornpaisarn & Kaewmungkun 2014 29	This study principally examines competition between three companies in influencing policy in an oligopolistic alcohol industry. The largest company was favoured by the taxation regime and largely succeeded in preventing the policy changes sought by the other two companies
Avery *et al*. 2016 40	A parliamentary enquiry recommended the inclusion of mandatory warning labels on all alcohol products against the preferences of industry actors. This did not translate into government policy, which continued instead with the existing voluntary labelling regime among other industry‐favoured measures on fetal alcohol spectrum disorders.
Zatonski *et al*. 2016 30	The public debate on alcohol policy in Poland was dominated by the views of the alcohol industry and other opponents of a spirits tax increase. Despite this, the tax was implemented, although the Government conceded that the spirits excise tax would not be increased again within the next 2 years
Savell *et al*. 2016 13	No data as not part of study design

Decisions at different stages of the policy process are not necessarily consistent, and can either favour, or go against, the interests of industry actors at different junctures (see Table 4). There are examples of competition between industry actors shaped largely by how policies affect different businesses and/or sectors of the industry. Throughout the studies examined here industry actors, nonetheless, have long‐term, well‐coordinated and well‐resourced strategies to advance their interests. Industry strategies appear transportable across national contexts, but are adapted flexibly to local issues and conditions, meaning that they do not take precisely the same form in every country (see Table 4). Although alcohol industry interests may prevail against public health interests due to long‐term influence on the policymaking environment, this does not mean that public health actors cannot, and have not, challenged successfully the framing and influencing activities of industry actors within the policy process.

## Discussion

The research literature on alcohol industry involvement in national policymaking has grown rapidly in recent years. This synthesis suggests that the two principal ways industry actors seek to influence policy are as follows: (1) to frame the contents of policy debates in a cogent and internally consistent manner; and (2) to adopt long‐term as well as short‐term approaches to managing threats to commercial interests that may arise within the policy arena by extensively building relationships with key actors, deploying resources in a variety of different organizational forms. Alcohol industry actors are thus highly strategic, rhetorically sophisticated and well organized and, for these reasons, present formidable competition to those seeking to reduce the societal and public health problems caused by alcohol.

The strengths of this study include rigorous systematic procedures for data collection and analysis and transparent reporting. Some review limitations reflect the limitations of the primary literature. Existing studies capture data that are relatively accessible. This is important, because it is challenging to observe the exercise of influence in policymaking 42. Without the aid of internal company documents, as have been available for tobacco companies, alcohol studies must use interview data to produce knowledge of what ‘working behind the scenes’ 24 actually involves. This means that existing knowledge at any point in time may only partially capture the phenomenon under study. This makes studies such as this one, where the totality of available evidence is examined before drawing conclusions, particularly valuable.

Existing studies are centred largely on policy controversies, and thus risk being more concerned with shorter‐term lobbying around specific events than with underlying processes that manifest themselves across national policymaking contexts and over the longer term. The review included seven reports from four discrete studies involving the current authors, and reflexivity considerations suggest that this presents a risk of giving too much weight to these studies. At the outset, it was unknown how far scholars in disciplines other than public health (e.g. political scientists) have investigated alcohol industry actor involvement in policy. This study identified only a small number of such studies 43, 44 which were ineligible for inclusion, as they lacked Methods sections. There is also a rich tradition of public health surveillance studies which lack methodological requirements but nonetheless contain valuable data on policy and politics, usually alongside data on commercial activities (for example, 2, 45). The focus on policymaking means that our findings in relation to framing, for example, omit studies of framing activities within the media, public relations or research rather than in policy *per se* 46, 47, 48. Also omitted are studies of the policy process lacking specific intent to study industry actors or presenting required data 11, 49, 50. A further limitation of this review is that we did not undertake a formal risk of bias assessment of the included studies.

It is appropriate to consider this study specifically in relation to the systematic review of alcohol industry attempts to influence marketing regulations 13 included here, while noting the risk of circularity. The two reviews examine largely different literatures due to differences in study designs, with only two primary studies 24, 26 included in both. Notwithstanding the differences in data sources and analytical methods used, there is a high degree of overlap in study findings. This is important, because it strengthens the existing evidence on the similarities in political strategy and tactics between the alcohol and tobacco industries.

The existing literature is based largely on individual high‐income Anglophone countries, and dedicated study of generalizability to other national contexts, particularly in low‐ and middle‐income countries, is lacking. We do not, however, only need more of the same kinds of studies identifying political activities and analysing discursive strategies. There are few cross‐national studies capable of illuminating precisely how industry actors adapt discursively and tactically to different institutional and cultural and policy contexts. Despite rhetorical efforts to differentiate alcohol companies from the tobacco industry, alcohol industry political strategies closely resemble, and are in some cases directly shaped by, tobacco industry actors. This suggests that it will be advantageous to use what is known about the tobacco industry to inform alcohol industry research agendas.

Future research needs to be ambitious to match the long‐term strategic character of industry actor involvement in policy and to be vigilant for developments in political organization, particularly as global concentration of production advances 51. Interviews are particularly useful sources of data collection on lobbying and related issues. Research of a more historical nature can address how industry actors develop their strategies over time, and address questions of how and why particular strategies are pursued. For example, why did social aspects organizations begin proliferating when they did, and how are these developments shaped by the changing structure of the industry? This particular example draws attention to the currently limited consideration of the commercial interests underlying policy preferences, and developing understanding rooted in political economy 52 should help to rectify this, as will integrating the emerging evidence‐base across corporate sectors 4.

Further research may also recognize that industry actors are one type of policy actor, and there is a need to situate them in relation to other actors within policy analytical studies. Similarly, keeping issues off the policy agenda is at least as important as delaying their progress, or shaping their specific content and direction once issues become identified as policy problems; hence the need for study of long‐term strategies. The framing of alcohol policy issues is in turn likely to be contingent upon wider discourses about the rights of national governments to intervene in the lives of citizens, the functioning of the market economy, the power of transnational corporations and where responsibilities lie for societal and public health problems.

This study shows that alcohol industry actors are involved in policymaking strategically to advance commercial interests, and this review builds upon previous findings across policy issues, across time, and throughout the world. We suggest that notwithstanding advances in understanding already made, this subject has been grossly understudied, given the importance to global health and the probable value of more advanced understanding of these issues. This study offers ideas for future directions in research on the basis of what is already known, and presents the evidence currently available that can be used to limit the deleterious effects of the political activities of the alcohol industry on population health and societal outcomes.

## Declaration of interests

None.

## Supporting information


**Appendix S1** MEDLINE search strategy.

**Appendix S2** Data extraction and preliminary coding.
