# Supplementary material for: Alcohol industry involvement in policymaking: a systematic review
Source: Addiction. 2018 May 6;113(9):1571–84. doi: 10.1111/add.14216 (PMC6100095; doi:10.1111/add.14216)
Supplement: Supplementary file 2 — Appendix S2 Data extraction and preliminary coding. [file ADD-113-1571-s002.docx]

**Appendix 2: Data Extraction and Preliminary Coding**

| Giesbrecht, N., et al. (2004). "Alcohol advertising policies in the United States: National promotion and control initiatives." Contemporary Drug Problems: An Interdisciplinary Quarterly 31(4): 673-710. | | |
| --- | --- | --- |
| **Category of Industry Activity** | **Summary of Data** | **Key quotations** |
| Policy approaches promoted (and arguments in favour) | Voluntary codes and self-regulatory regimes for advertising and marketing (to ward off mandatory regulations).  Prevention and harm reduction programmes.  Television advertising for spirits.  Promotion of the health benefits labels of wine. | Advertising codes adopted by alcohol trade associations signal the sensitivity of some producers to pressure about this issue.  Alcohol industry efforts in the direction of "responsible  advertising" include voluntary codes by trade organizations  that discourage sexually provocative advertising and  promotion to persons under the legal age of purchase.  Diverse interests develop and sponsor prevention and harm-reduction messages directed at pregnant women, problem drinkers, and children. There is a strong alcohol industry presence in messages against drinking and driving, including the promotion of designated drivers, alternative forms of transportation, zero tolerance for underage drinkers, and school programs. |
| Policy approaches opposed (and arguments against) | Advertising restrictions since, from industry’s perspective, people already know the risks and self-regulation works; warned of unintended consequences (especially for poorer people) and potential breaches of the constitutional right to free speech. | They argued that the proposed *SAFE Act* was unnecessary because the public was already aware of the hazards of drinking and because broadcasters, advertisers, and alcohol companies had advertising codes. |
| Industry self-definition/ positioning as a policy actor |  |  |
| Industry definition of other policy actors |  |  |
| Unity/cleavages across industry | Spirits advertising not supported by other sectors of the industry.  Underage Drinking. | This issue prompted temporary realignment among interest groups. The repeal of the voluntary ban on the part of the Distilled Spirits Council of the United States (DISCUS) was viewed unhappily by the other sectors of the alcohol industry (brewers and vintners), which had hitherto enjoyed exclusive access to broadcast advertising outlets. Their concern was that permission to advertise spirits on television would cause television to be flooded with alcohol advertisements,  resulting in a public reaction against not only spirits but also  beer and wine advertising.  The alcohol industry appears quite ambivalent, split and  fluctuating on this issue. On the one hand, in general it  cannot appear to condone underage drinking. Yet industry sources have at times argued for relaxation of the now national minimum legal drinking age (LDA) standard of 21 years old. |
| Influencing activities | Delivering ‘policy goods’ to government through the offer of self-regulatory regimes in advertising/ promotions oversight.  Opposed what they claim is flawed evidence for unflavoured measures.  A common industry argument is that "advertising affects only market share and does not increase the overall consumption of alcoholic beverages." |  |
| Channels of policy influencing activity | Trade Associations.  Key advocate on industry position was Senator Strom Thurmond (Main Sponsor of SAFE) during Congressional hearings and at key stages of process (after death of his daughter). |  |
| Targets of policy influencing activity |  |  |
| Position of the industry in policy process |  |  |
| Success/ effectiveness of industry influencing activities. | Labelling.  SAFE Act (Advertising and Marketing).  Comprehensive Alcohol Prevention Act failed to come to a vote but no evidence presented of industry responsibility for this.  Broadcast Advertising of Spirits  Ads removed under widespread criticism and pressure.  Health benefits labelling for wine. | In September 1988, the Senate Commerce Committee unanimously approved the warning labels bill. The regulation initially provided so much latitude in design, such as colors, alignment, and position, that the alcohol industry could render the labels neither prominent nor easily read; in 2000, the regulation was tightened with more specific design requirements. This outcome would seem to have been less  intrusive to the alcohol industry than legislated controls on alcohol advertising and may also have given the industry some perceived protection from product liability, given the concurrent tobacco litigation.  In general, the 1989 warning-label legislation was considered a victory, even if a muted one, by public health advocates. However, some key players observed that it offered alcohol producers and distributors a possible escape from more onerous (and perhaps more effective) restraints down the road.  At this juncture, however, broadcast and alcohol  industry groups offered to run a public-awareness campaign  as a voluntary alternative to legislation. This offer was  accepted by Thurmond, despite initial misgivings, and he withdrew the bill. The public-awareness campaign became known as STAR (Stations Targeting Alcohol Abuse Reduction).  However, the 1995 revision of the U.S. Dietary Guidelines  *omitted* the claim that alcohol consumption "has no net  benefit" and "is not recommended," and *included* the statement "Alcoholic beverages have been used to enhance the enjoyment of meals by many societies throughout human history". Thus the wine industry's lobbying had been successful.  After several years' delay, the follow-on organization to  ATF (now absent firearms), the Alcohol and Tobacco Tax and  Trade Bureau (TTB), issued final rules in February 2003  allowing health statements on wine labels but with strict  requirements to be met by producers. |

| Greenfield, T. K., et al. (2004). "The alcohol policy development process: Policymakers speak." Contemporary Drug Problems: An Interdisciplinary Quarterly 31(4): 627-654. | | |
| --- | --- | --- |
| **Category of Industry Activity** | **Summary of Data** | **Key quotations** |
| Policy approaches promoted (and arguments in favour) | Industry arguments favour targeted policy measures and individual responsibility versus whole population measures. | On the industry side, the evidence in our transcripts suggests the following framing of the problem: "low general social control, individual responsibility, punish the few rotten apples." Industry informants tend also to look  at problems like excessive drinking by certain groups as  manifestations of larger socio-cultural ills and are supportive  of childhood educational interventions. Most industry  spokesmen appear unconcerned about the considerable literature indicating little effectiveness of such individual and school-based approaches. |
| Policy approaches opposed (and arguments against) |  |  |
| Industry self-definition/ positioning as a policy actor | Highlight the importance of the industry as a generator of tax revenues and politicians were widely receptive to this. |  |
| Industry definition of other policy actors | Attempts to position public health actors as extremists and/or prohibitionists. | The allusion to alcohol's being "here to stay" is interesting,  since no one we interviewed in the public health policy  community advocated a return to prohibition. Part of the  rhetorical positioning of industry groups is to imply that their policy adversaries are extremist neo-prohibitionists. |
| Unity/cleavages across industry | Pressure exerted on industry actors who may support public health measures not to break rank and to support the interests of the industry more widely or specific sectors of the industry. | However, retailers acting through their national organizations threatened to "blackball" the producer's brands if they opposed the action to defeat the bill.  Thus, via the power of the marketplace, industry groups,  though often in competition with one another and at best only loosely allied, have a way of exerting political discipline  within, and sometimes across, sectors. |
| Influencing activities | Industry actors also have long term ‘intimacy' with Capitol Hill legislators’. Contacts with the party leaderships were particularly important and effective.  Setting the tone or mood music for the policy environment is a key mechanism for creating conditions for a favourable policy environment. There is a cumulative effect of even unrelated events and interventions which build momentum towards policy changes and can lead to a sense of inevitability that something is going to happen.  There was a need ‘to keep plugging away’ on pet issues to move them onto the agenda and develop policy responses. | Seizing opportunities to force events and to help alter  the national mood is mentioned in the transcripts, as is the notion of building momentum for change on the Hill creating an expectancy that "something is going to happen." |
| Channels of policy influencing activity | Trade associations such as the Beer Institute, which employ former congressmen as executives and lobbyists. Have been highly effective in marshalling votes in the Senate and House.  Citizens movements and ‘grassroots’ organizations (see below).  Having ‘champions’ in key administrative or policy making functions were key mechanisms of influence.  Politicians with industry undertakings in their districts. | Many members have large  breweries, distilleries, or vintners in their states or districts, and they are kept appraised by the lobbyists for economic and commercial interests on how the well-being of these companies can translate to votes via the number of jobs directly and indirectly associated with purveying the product, from production and distribution through retail, as well as in  advertising and broadcasting. |
| Targets of policy influencing activity |  |  |
| Position of the industry in policy process | Widespread perceptions amongst policy actors that industry resources lead to access not enjoyed by public health actors. Some evidence to support this cited by authors. This was linked to the campaign financing system that meant politicians needed to take tainted money with quid pro quos. To counter this PH advocates needed widespread public support. | ‘The idea that industry interests have money and access was contrasted to perceptions of the advocates that they often had superior abilities to muster a groundswell of grassroots and  constituent organizations' political activity-not only letter writing campaigns but also calls by personal friends of  members well connected in their districts:  . . . the only way to combat corporate interests is through overwhelming public support that is perceived by politicians to ultimately generate votes. It's just the only thing we have; we don't have PACs [Political Action Committees], we don't have this, we don't have their access.’ |
| Success/ effectiveness of industry influencing activities. |  |  |

| Bond, L., et al. (2009). "Title: Access to confidential alcohol industry documents: From 'Big Tobacco' to 'Big Booze'." Australasian Medical Journal 2(3). | | |
| --- | --- | --- |
| **Category of Industry Activity** | **Summary of Data** | **Key quotations** |
| Policy approaches promoted (and arguments in favour) | Remedial programmes on drink driving and stricter enforcement of blood alcohol laws.  Industry run youth education programmes. | Further, education and prevention programs have been developed by PM [Philip Morris] and the BI [Beer Institute] in an attempt to  minimise regulation of alcoholic beverages and to present an image of corporate social responsibility. The MBC [Miller Brewing Company] have “…a multi-faceted alcohol-awareness and education program to remind consumers of the risks and to promote responsible drinking”. |
| Policy approaches opposed (and arguments against) | Tax increases on beer products, restrictions on marketing/advertising, labelling requirements.  Tried to frame debates in terms of a small minority of problem drinkers, thus rendering tax increases an inappropriate responses. They also make equity arguments (i.e. that taxes hit the least well off hardest).  Opposed advertising and marketing restrictions, citing a first amendment defence. They deny link between advertising and harms or increased consumption.  Claimed advertising doesn’t appeal to minors.  Advertising only function is brand switching amongst legal adults. They make equity arguments again here.  Oppose product warning labels  Oppose reduction of blood alcohol content (BAC) laws and increases in minimum purchase age. Argue most people can drive safely after moderate drinking (as opposed to being ‘drunk’).  Denied the link with domestic violence and any industry responsibility for this. | Key alcohol related concerns included: proposed legislative and regulatory controls such as tighter restrictions on advertising, marketing and sales; strong enforced labelling including health warnings; blood alcohol content lowering; and measures to increase taxes and legal drinking age. Further concerns stem from community, health and social issues and pressure from health groups regarding harms from product use. These included underage drinking, “binge drinking” [sic], drunk driving, alcohol abuse and domestic violence.  Miller opposes excise tax increases based on social costs. Our industry is already paying its fair share of government coffers…Since the majority of people who drink do so responsibly, it is unfair to penalize the majority to pay for the actions of a few.  Numerous credible studies conclude that advertising does not cause alcohol abuse; therefore ad bans will not stop it. Studies conducted by government and independent researchers conclude advertising affects brand performance, not consumption or abuse.  Doesn’t Miller’s advertising appeal to underage people? While we understand your  scepticism, sound research shows that advertising has a negligible effect on youth  consumption; on its own, advertising doesn’t make young people drink.  PM argue that the company advertises its beer to encourage consumers of legal drinking age to select the  Miller brand over other brands. It also aims to “remind drinkers about the importance of consuming products responsibly”  There is no empirical evidence that warnings would have any effect on the drinking  patterns of Americans of any age, especially among alcohol abusers. Worse, these types  of warnings could undermine the credibility of other government campaigns to provide information about serious risks which are not commonly known.  Will PM support a proposed national goal of uniform state BAC standard of .08? No. Reducing the BAC will not get to the root of the drunk driving problem (repeat offender with a BAC of .17 and higher). Remedial programs and stricter enforcement [are] more likely to be effective.  It is not the product that  causes violence, but the individual user: “too many domestic abusers use alcohol as an excuse not to  change”. PM have consulted ‘experts’ who conclude “alcohol does not cause the battery – any more  than stress, illness, or being ‘out of control’ does. Domestic violence, they tell us, is a learned and chosen  behaviour intended to control an intimate partner” |
| Industry self-definition/ positioning as a policy actor | Concern about drinking becoming socially unacceptable, much of this centred on public concerns about ‘binge drinking’. Promoted separation of responsible and abusive drinking. The latter is confined to a problematic minority of drinkers or addicted ‘alcoholics’ (which they claims is around 5.5% of the population). | The alcohol industry  must also reassure consumers that “beer consumption is socially acceptable”.  “As long as the public continues to view ANY consumption of alcohol and ABUSIVE use of alcohol as two different subjects – a view the  brewing industry has successfully encouraged – the majority of the anti-alcohol agenda will not be met  with much success”. |
| Industry definition of other policy actors | Feared new sources of arguments for regulation and the rise of ‘neo-prohibitionists’. | A five year plan devised by MBC (1992–1996) reflected these concerns. This document acknowledged a movement of social attitudes toward the moderation of consumption of alcohol, along with growing “neo-prohibitionist legislative  sentiment”. Predictions included continued pressure to increase excise taxes, extend warning label requirements and increase advertising restrictions. |
| Unity/cleavages across industry |  |  |
| Influencing activities | MBC to learn from tobacco experience (PM).  Driven purely by commercial interest, they promote less effective alternative policies and highlight alleged negative externalities.  Consistently question the evidence base for unfavoured policies and present favourable alternative evidence. | These moves towards stronger controls have encouraged internal collaborations  between the PM divisions.  The overall approach by WRA [Worldwide Regulatory Affairs] was “…to fight aggressively, with all available resources, against any attempt, from any quarter, to diminish our ability to manufacture our products efficiently, and market them effectively”.  The research thus far has failed to identify any evidence of concern by the drinks industry about the many  problems caused by harmful and hazardous drinking other than as they relate to the industry’s own interests and profitability. |
| Channels of policy influencing activity | Trade associations named:  Beer Institute.  National Beer Wholesalers Association. | “We will oppose  excessive tax increases by combining our efforts with various beer/alcohol beverage organizations, such  as the Beer Institute and the National Beer Wholesalers Association, as well as grass roots organizations” |
| Targets of policy influencing activity | Policy makers and wider public. |  |
| Position of the industry in policy process | Links between alcohol and tobacco industries. |  |
| Success/ effectiveness of industry influencing activities. |  |  |

| Bakke, O. and D. Endal (2010). "Vested interests in addiction research and policy alcohol policies out of context: drinks industry supplanting government role in alcohol policies in sub-Saharan Africa." Addiction 105(1): 22-28. | | |
| --- | --- | --- |
| **Category of Industry Activity** | **Summary of Data** | **Key quotations** |
| Policy approaches promoted (and arguments in favour) | Underplay scale of alcohol problems; emphasise positive aspects of alcohol and protect commercial interest of a legal industry.  Emphasise the effectiveness of public awareness raising and communication of harm.  Review of blood alcohol content.  Advocate self-regulatory regime for alcohol availability, marketing and promotions.  Individual framing of at risk populations.  Wanted evidence based approach to provision of alcohol treatment services applicable to LMIC context. | This and other documents emphasize specifically that: ‘[t]he Government acknowledges that alcohol enjoys popularity and a place of significance in Basotho society. Alcohol when used in moderation has a positive role to play in socialisation and the industry is a major contributor to the economy of Lesotho’. One of their key guiding principles enshrines ‘the right of the alcohol industry to conduct legitimate and legal business in a responsible way’. Although the policy documents recognize the negative impact of alcohol use, the availability of alcohol seems to be one dominant theme: ‘the need to protect the reasonable expectations of adult citizens of Lesotho to purchase and consume alcohol in a safe and well regulated manner’.  The last part of the draft guidelines for implementation places the responsibility for implementation of the policy with a ‘National Alcohol Council’, on which representation  is reserved for the industry:  Whatever the content of these discussions in the workshops,  the draft national alcohol policies take essentially the  same approach proposed in ICAP’s publication [International Center for Alcohol Policies] *Drinking in Context*, where the emphasis rests upon the need to manage drinking patterns and strengthen industry/ government/public health partnerships. |
| Policy approaches opposed (and arguments against) | Ignores international research consensus and World Health Organization (WHO) best buys.’ | The draft policy documents are devoid of any reference to *Alcohol: No Ordinary Commodity* or other compilations of the international evidence base on  alcohol prevention developed by independent alcohol researchers working on behalf of the WHO. Only a few of the best practice recommendations are included in the set of six policy priority areas given in the documents (five policy areas in the Uganda document). |
| Industry self-definition/ positioning as a policy actor | Key stakeholders | The policy drafts reflect a consistent emphasis on the role of alcohol in society and the legitimacy of industry participation in the development and implementation of national alcohol policies |
| Industry definition of other policy actors |  |  |
| Unity/cleavages across industry |  |  |
| Influencing activities | Provision of industry drafted documents which formed the textual basis of alcohol policies of 4 African governments (Lesotho,  Malawi, Uganda & Botswana).  ‘Consultation’ with policy makers in the development and implementation of policy.  Creation of new bodies with industry presence to implement policy.  Organisation of conferences and workshops attended by industry and policy makers. | The draft policy documents we reviewed describe their origin as having ‘been developed as a result of broad consultations including two national symposia attended by senior representatives of Government Agencies, Non-Governmental Organizations, the beverage alcohol industry and representatives of civil society’. The properties information of two of the MSWord documents indicated that the ‘author’ was ‘mramsay’ from the ‘company’ SABMiller Africa Asia, possibly Mitch Ramsay, Policy and Issues Manager, SABMiller Africa. His later comment that ‘at the invitation of the workshop attendees, I was invited to prepare a record of the workshop policy proposals in local policy format’ may explain this (Ramsay, M., unpublished observations).  The initiative to develop an alcohol policy came from the Ugandan parliament and the task was entrusted to the Ministry of Health. The alcohol industry came on board only later. In the other cases the initiative seems to have come from the drinks industry.  All four documents have the same core of policy measures  and some key formulations that we would expect the alcohol industry wants to see included. The Lesotho  and Malawi documents are almost identical. The Botswana document is also very similar to the two discussed above. Some sections were moved, some removed and other changes had been incorporated. The Uganda document differed somewhat more from the  three others.  The documents describe that senior representatives of  government agencies, NGOs and representatives of civil  society groups have been invited to attend workshops and consultations. Reportedly, the workshops were facilitated by Mitch Ramsay of SABMiller and Mr Keith Evans. |
| Channels of policy influencing activity | SAB Miller executives, possibly Mitch Ramsay.  Keith Evans, South Australia Dept. of Health and Consultant to ICAP. |  |
| Targets of policy influencing activity | Governments of 4 countries above (potentially other African countries). |  |
| Position of the industry in policy process |  |  |
| Success/ effectiveness of industry influencing activities. | Industry authorship of 2 national alcohol policy draft documents and cut and paste into 2 others. Strong and ongoing process of engagement and long-term relationship building established. | The enormous market power exercised by the alcohol companies can translate easily into political power. The policy development processes and the proposed policy drafts provide insights into the working methods of some of the multi-national drinks companies. |

| Bond, L., et al. (2010). "Selling addictions: Similarities in approaches between big tobacco and big booze." Australasian Medical Journal 3(6): 325-332. | | |
| --- | --- | --- |
| **Category of Industry Activity** | **Summary of Data** | **Key quotations** |
| Policy approaches promoted (and arguments in favour) | Public awareness and education programmes (especially on youth drinking and drink driving). | A document from the  Beer Institute titled ‘Preventing Alcohol Abuse’ stated that:  “The industry alone has spent over $250 million over the  past decade to fund research, public safety, education and  prevention campaigns to curb alcohol abuse”. Examples of  alcohol industry programs included a cooperative effort by brewers Anheuser-Busch, Miller, Coors and Stroh to  promote responsible consumption by adults, and to “fight drunk driving and underage drinking” |
| Policy approaches opposed (and arguments against) | Common issues between tobacco and alcohol sectors.  Many of these issues are the same as those identified in Bond et al 2009 (e.g. tax rises marketing restrictions, including youth, warning labels).  Here, the additional focus is on product placement and on targeting minorities. | Alcohol and tobacco industries are faced with legislative  restrictions such as: advertising constraints; labelling regulations; supply and retail restrictions; and are  susceptible to tax increases and disapproval from health  advocates.  Similarly, there is evidence from the archives that PM has  attempted to target market its beer products to minority  groups. An inter-office correspondence document from MBC acknowledged: “A disproportionate share of total  Miller volume is purchased by minority consumers”. This  correspondence, with the heading ‘Minority Relations’  outlines the need for Miller to place emphasis on developing stronger relations with minority organisations and community leaders:  ‘Existing programs of minority print media advertising, community involvement and  cultural support within the black community will be enhanced. New or expanded  programs targeted to the Hispanic community in key market areas are in  development, and will include greater involvement with Hispanic print media, support of key business associations and organizations, and an increased visibility for Hispanic directed promotional marketing efforts’.  ‘Miller responds to requests from movie companies which desire to have authentic products depicted in their movies as a natural part of a scene. What Miller pays for is to have our brand identified over someone else’s’. |
| Industry self-definition/ positioning as a policy actor |  |  |
| Industry definition of other policy actors | Dismissed the concerns of industry critics, claiming public health advocates are arbitrarily seeking something to get angry about. These people are often driven by a moral agenda. | ‘Many of the threats to us, P.M., arise from concerns which have lost touch with common sense and reality. People (and politicians) do need causes, and in a world  which is generally more peaceful and affluent than ever before, there’s a shortage  of big causes. That’s why we hear so much about really rather little causes: smoking,  drinking, dietary hazards…’. |
| Unity/cleavages |  |  |
| Influencing activities |  |  |
| Channels of policy influencing activity | Strong links between PM tobacco and alcohol divisions, which used the same public relations/ lobbying personnel and deployed similar arguments on key policy issues. | To promote “cross-company synergies”, Worldwide  Regulatory Affairs (WRA) was initiated by PM to take “a  liaison role, bringing the various players across companies together and creating the networks and communication vehicles” to share information regarding management of issues. A brief to the PM board described the role of WRA:  ‘We participate in a broad coalition of organisations committed to defending  commercial free speech, which includes member companies of the tobacco industry, allied industries like the liquor industry, the advertising industry, industry trade  groups…’. |
| Targets of policy influencing activity |  |  |
| Position of the industry in policy process |  |  |
| Success/ effectiveness of industry influencing activities. |  |  |

| Miller, P. G., et al. (2011). "Vested interests in addiction research and policy. Alcohol industry use of social aspect public relations organizations against preventative health measures." Addiction 106(9): 1560-1567. | | |
| --- | --- | --- |
| **Category of Industry Activity** | **Summary of Data** | **Key quotations** |
| Policy approaches promoted (and arguments in favour) | Targeted, information and education based approaches (e.g. social marketing campaigns) versus whole population measures, although no evidence is cited in support of this (the evidence suggests complete independence is needed for the providers of social marketing for it to work). | Anderson and others have identified a number of common positions which support the interests of their funders, the alcohol industry. These include: (i) focusing on pathological or ‘abusive’ drinking patterns, rather than industry practices; (ii) avoiding research into effective measures, such as pricing and availability controls; (iii) trying where possible to recruit young researchers; (iv) advocating industry self-regulation; and (v) promoting ineffective social marketing practices.  Both Drinkwise employees were strongly in favour of substantially increasing the level of ‘social marketing’ campaigns. Mr Watters wrote that: ‘Some of the actions  identified in the NPHT [National Preventative Health Taskforce] Report provide the opportunity for the design and delivery of new thinking such as addressing the cultural place of alcohol via carefully planned, targeted and research-based social marketing’. The Drinkwise submission went on to report that: ‘Accordingly, DW engaged in a mass advertising campaign, and the provision of support and advice to parents and young people alike, from a panel of independent experts’. |
| Policy approaches opposed (and arguments against) | Drinkwise employees’ responses to the consultation opposed the measures set out in the NPHT white paper (see quotation), but no evidence is cited for their dismissal of the ‘broad brush approaches’ contained in the White paper.  Opposed restrictions of sports sponsorship and advertising  Opposed pricing interventions. | The NPHT White Paper ranked interventions in order of priority: (i) regulating physical availability; (ii) taxation and pricing; (iii) drink driving counter-measures; and (iv) treatment and early intervention. Other areas identified by the NPHT that have potential for effectiveness include: altering the drinking context, regulating promotion, and well-funded, sustained public education. Mr Watters’ response to the report’s recommendations was that: ‘DW believes that some of the action items proposed [in NPHT report] to achieve the key priorities represent old thinking and lack a strategic and holistic approach’. Dr Dann’s submission proposed that: ‘The Taskforce has placed an over reliance on the use of the broad brush stroke approach of regulation’.  In the official Drinkwise submission, Mr Watters argued against evidence-based practice, such as increasing alcohol price and reducing its  availability, stating that Drinkwise did not recommend ‘fiddling with alcohol tax’, because it was ‘old thinking’. |
| Industry self-definition/ positioning as a policy actor | Emphasis on Drinkwise as a key aspect of industry CSR activities  Drinkwise defined as ‘an evidence based organisation’  Legitimate funder of research which is promoted in responses | For example, the Distilled  Spirits Industry Council of Australia (DSICA) wrote: ‘The work being undertaken currently by Drinkwise  Australia to promote sensible drinking through cultural change is but one example of the commitment of the  alcohol industry within Australia to the promotion of a positive and healthy lifestyle’  ‘Over the past 3 yrs, DW [DrinkWise] has funded research by leading academics across a number of Australian Universities, e.g.; the seminal research on the  cultural drivers impacting upon young people and alcohol, by Prof Ann Roche, Flinders University has  identified a range of key influences on Gen Y, indicating current policies have little to no impact on young, single, upwardly mobile, brand conscious, highly educated, affluent and technologically astute drinkers.’ [Chis Watters (CEO of Drinkwise)] |
| Industry definition of other policy actors |  |  |
| Unity/cleavages across industry |  |  |
| Influencing activities | Use of social aspects/public relations (SAPROs) (funded by the industry with varying degrees of industry control in their management structures) to promote industry perspectives whilst feigning independence.  Research is also undertaken by Drinkwise and cited in consultation responses to support favourable positions. | ‘DrinkWise Australia is an independent, not-for-profit  organization focused on promoting change towards a healthier and safer drinking culture in Australia’. However, this claim has been challenged, particularly because in the ‘balanced board’ of six industry representatives and six ‘community’ members, some of the community members have financial ties to the alcohol industry. |
| Channels of policy influencing activity | SAPROs (principally Drinkwise Australia in this case).  All responses to Australian Government consultation on the National Preventative Health Task Force) by industry actors referred to Drinkwise.  Also submissions were provided to the consultation by Drinkaware employees. |  |
| Targets of policy influencing activity | Government via consultation responses |  |
| Position of the industry in policy process |  |  |
| Success/ effectiveness of industry influencing activities. |  |  |

| Holden, C., et al. (2012). "Cleavages and co-operation in the UK alcohol industry: a qualitative study." BMC Public Health 12: 483. | | |
| --- | --- | --- |
| **Category of Industry Activity** | **Summary of Data** | **Key quotations** |
| Policy approaches promoted (and arguments in favour) | Companies and trade associations adopted differing positons on MUP (minimum unit pricing) and also tax issues which are cited as examples of cleavages. Anti-MUP position became the dominant industry voice, however. | ‘Molson Coors is open around below cost selling and minimum pricing – none of our competitors are. We took a conscious decision about eighteen months ago to increase our prices in the market, and to cut volume, and that has helped us. . . we think that’s part of actually changing beer. . . the beer agenda. Since then some of our competitors have followed us on that, and others haven’t; so very different strategies and beliefs.’ |
| Policy approaches opposed (and arguments against) |  |  |
| Industry self-definition/ positioning as a policy actor |  |  |
| Industry definition of other policy actors |  |  |
| Unity/cleavages across industry | Highly fragmented, but able to co-operate/ coordinate activities on issues of mutual concern  Producers v retailers  On v off-trade  Beer v spirits  Cider v vodka  Beer v cider  Brewers v other brewers  Premium brands v ‘commodity products’  Small retailers v large supermarkets; Different firm/ sector positions and thus alliances/ cleavages will vary between issues (i.e. tax v MUP)  Internal cleavages also exist within trade associations (e.g. BBPA with pub operators and producers).  Tesco and Morrison’s (which moved to support MUP) versus other supermarkets (which opposed it).  Close, often personal connections between operators (companies and trade associations) across the industry. Trade Association Directors group have regular meetings.  Policy makers use industry cleavages to push through policy agendas. | ‘This is a very, very fragmented industry. No one likes each other very much; the on-trade hates the off-trade, wine and spirit and beer companies they’re all arguing about various things.’  ‘We go through the collective organisations because we also believe [. . .] we would like to have one view as an industry because this is the better way to do it and this is also why associations are there, to  represent us. And even if we don’t agree with everything that others say, it’s better to have a common view than to come up as a split kind of tribe.’  ‘And sometimes it’s also quite powerful for companies to take a different position to the rest of the sector because they can say to government and people, ‘Look, we’re the responsible guys here. It’s those others you’ve got a problem with’ in lots of areas that they’d be dealing with them. So sometimes our members like to have a different position to everybody else, to say that, ‘We’re the ones who are differentiating and we’re different. So we’re up here and they're down there somewhere’.  Given that many organisations are members of multiple trade associations, the contacts between their representatives are frequent. This can lead to the formation of strong and enduring personal relationships between the individual representatives of different organisations within these forums.  Policy makers may also find it useful to exploit the cleavages between certain actors in order to drive forward their policy agenda. As a former Minister of Public  Health put it:  So I mean the trade associations were helpful, but do you know sometimes just trying to find, I think in this area, trying to find a leader in that sector who’s prepared to break from the pack and do something different, or be prepared to think about things  differently, is actually a really… I think that’s a really good win if you can do that. |
| Influencing activities |  |  |
| Channels of policy influencing activity | Trade associations, individual company representations, SAPROs.  Smaller companies act via trade associations.  Larger companies try to do this too and see advantage in unity of voice, but can and do act alone when there’s an advantage.  Industry actors often take a co-ordinated approach allowing some actors to serve as the principle spokesman or lead organisation on a given issue (e.g. MUP). | A representative from Alcohol Focus Scotland, for example,  recalled that it was often the PG [Portman Group]– along with trade associations such as the WSTA [Wine and Spirit Trade Association] – who would represent the industry perspective in the public debates on MUP in which she participated. Similarly, the PG felt it appropriate to make a submission to the Scottish  Government’s consultation on the Alcohol Etc (Scotland) Bill. The appearance of autonomy from the industry is important to the function of the PG. It gives the organization a credibility and status not afforded to trade associations whose remit is more overtly to represent the interests of its members. Consequently, the PG has been afforded an important role not just in the regulation of alcohol marketing, but was given great prominence within previous governments’ alcohol strategies.  This manifests itself in a division of labour between trade associations, whereby those with a particular interest in certain issues, or a specific expertise or geographical focus, will take the lead on a given issue. The clearest example of industry coordination of this type occurred in Scotland where the Scotch Whisky Association (SWA) took the lead on the issue of MUP, reflecting their specific knowledge of Scottish politics, the economic importance of the whisky industry in Scotland and the emblematic nature of whisky for Scottish identity. |
| Targets of policy influencing activity |  |  |
| Position of the industry in policy process |  |  |
| Success/ effectiveness of industry influencing activities. |  |  |

| Holden, C. and B. Hawkins (2013). "‘Whisky gloss’: The alcohol industry, devolution and policy communities in Scotland." Public Policy and Administration 28(3): 253-273. | | |
| --- | --- | --- |
| **Category of Industry Activity** | **Summary of Data** | **Key quotations** |
| Policy approaches promoted (and arguments in favour) |  |  |
| Policy approaches opposed (and arguments against) | Ideological opposition to market intervention (see above).  Claimed MUP would be ineffective and have adverse, unforeseen externalities, especially in terms of the economic impact. | This involved framing the nature of alcohol-related harm in very narrow terms, focusing on social order issues and an allegedly small group of hazardous and harmful drinkers. Policies, it is argued, should focus on these groups through targeted interventions  and should aim to change the culture around alcohol through public awareness and educational campaigns. MUP, in contrast, is seen as failing to target these problematic  drinkers and as unfair since it affects the vast majority of the population who drink normally and responsibly. Furthermore, it may have a detrimental effect on the Scottish economy since whisky is such a vital component of the economy and a provider of employment. |
| Industry self-definition/ positioning as a policy actor | The part of the industry opposed to MUP emerged as the dominant industry voice and appeared at time to be the industry voice. | Although the industry was divided on the issue of MUP, those opposed to the proposed measures on price seemed more vehement in the defence of their interests and more vociferous in their objections than those industry actors that were prepared to accept it. This may reflect in part the stakes at play for the largest producer organisations. |
| Industry definition of other policy actors |  |  |
| Unity/cleavages across industry | Some evidence of cleavages between groups on MUP (see above). |  |
| Influencing activities | Counter-framing efforts to oppose public health attempts to reframe debate towards health/ whole population measures  Key industry objective was to avoid policy spillover (to other domains in the UK and beyond) and to prevent real world empirical evidence of MUP’s effectiveness.  Moreover the introduction of MUP would make lobbying efforts more difficult in other (developing ) markets.  Lobbying of Members of the Scottish Parliament (MSP) often focussed on effects on MUP in their won constituencies.  Extensive media campaign against MUP at key stages in the policy process to get public/ voters opposed to policy and thus pressure MUPs to oppose.  Attempts to shift the locus of decision making from Westminster to London (by arguing for taxation as a preferable alternative to MUP). | ‘We know that Northern Ireland, Ireland, Wales and places across the world are actually looking at this as a policy option. Yeah, I suspect that’s part of . . . they had a concern that it would work basically, that we would get it in place and be able to demonstrate after a few years that we were actually achieving the reductions in consumption and harm that our modelling suggested would happen. I think that’s the concern, that it would be seen as successful and therefore it would be copied.’ (Civil Servant, November 2010).  ‘If I was working for [a large alcohol producer] lobbying the Chinese government or the Indian government or whoever it might be, the last thing I would want is my own country to be regulating the alcohol industry and giving the impression that the industry can’t be trusted to regulate itself. They want to be in these countries saying, ‘We’re responsible. You let us deal with promotion and responsible messaging and everything else’. I think that’s maybe part of the opposition to minimum pricing and regulations on promotions. The message that sends to other countries, that the industry must need regulating. Flashes of that came through from some discussions that we had with the industry. I think we were a couple of months into the process before we realised that this is really not just about minimum pricing in Scotland. There’s much  bigger issues that they’re concerned about here.’ (Civil Servant, November 2010).  ‘In addition to that there were opposition members who have bottling plants in their constituency and those individual companies were in to see them. In fact I passed a few colleagues from industry in the corridors . . .They were heading up the Labour corridor to see somebody there. Yeah, there’s no doubt that that was quite a big part of it. To what extent that informed the position taken by Labour and the Liberal Democrats we’ll never know. But yeah that was part of the process, absolutely.’ (Civil Servant, November 2010)  ‘What then happened was the Scotch Whisky Association and the Wine and Spirit Trade Association both commissioned public affairs consultancies. The brief that they sent round the public affairs consultancies that were tendering for the work found its way into the press and basically what was written in the brief was that the task that the public affairs consultancies were charged with was to remove minimum pricing from the alcohol legislation before it goes into stage one.’  As the debates around MUP continued, and the determination of the Scottish  Government to address the issue of price became more apparent, some industry actors that initially opposed MUP began to shift on the issue, to the point where they were prepared to consider a ban on sales below the level of Duty plus VAT [Value-Added Tax] as an apparent alternative approach. Off-trade retailers, including the largest high street supermarkets – whose business models would be affected by the introduction  of MUP – and certain producer organisations came to advocate this approach. Even the SWA, which had voiced strong reservations about price interventions, began to consider a ban on below-cost sales as an alternative to MUP, albeit with some reservations. |
| Channels of policy influencing activity | Use of overseas actors (including government to lobby against MUP): Irish Spirits Association and Chilean Government.  Favourable MSPs brought industry messages into key policy making forums.  SWA as key conduit of industry messaging and lobbying activities. | ‘We also had a fantastic exchange of correspondence with the Chilean Government who were most concerned about the impact on their wine. That was an industry trade association, you could tell I think. One of the letters didn’t even make an effort to disguise the wording. It was just a cut and paste from some briefing notes. We had prolonged exchange between the First Minister and the Chilean Ambassador.’ (Civil  Servant, November 2010)  ‘You know I’ve witnessed in committees quite literally some of the committee members almost reading out word for word the questions that some of these alcohol companies and retail companies have actually been putting out in their press releases; you know, and you’re left saying to yourself, you know, what is the point of this if you’re just allowing yourself to be completely manipulated by these organisations.’ (MSP, September 2010).  ‘The interesting thing though is we’ve always had our suspicions about the position of some of these trade bodies because their membership make an awful lot more than whisky. What we’ve found in this debate is that the Gin and Vodka Association have  been completely silent. Now their members would be affected by minimum pricing. There are cheap vodka brands which would have been hit by the 45 pence minimum  price. But other than initial consultation response, they just didn’t have any part in this debate at all which we found quite odd. That made us wonder whether the other trade organisations had basically said, ‘Well just leave this to us, we’ll deal with this.’ The arguments which were given a whisky gloss were actually more about other products within the same companies.’ (Civil Servant, November 2010) |
| Targets of policy influencing activity | Shifting, reactive strategy in response to contours of devolution, the change in government (lack of political connections with SNP and minority administration) from focus on Ministers/ civil servants/ governments MSPs to opposition MSPs. | The shift in direction of alcohol policy under the SNP minority government from 2007 presented a threat not simply to the corporate interests of certain industry actors but to their entire corporate affairs strategy. The launch of the consultation on the SNP’s proposed legislation was met with a concerted campaign by industry actors, led by the SWA, to remove MUP from the alcohol Bill. The determination of the SNP to press ahead with MUP, and the reliance of the Scottish government on support from opposition parties in order to pass its legislation, led to a shift in lobbying activities from ministers and civil servants to MSPs and an attempt to shape the broader public perceptions of MUP through the media.  ‘But what they decided to do was try and pick off individual ministers, regardless of what their portfolio was. [. . .] They came in, they argued their case to the minister responsible, she wasn’t persuaded. Then they just went through week after week, going through the same process of making the same points with different ministers. The organisations like the Scotch Whiskey Association, they would generally get a meeting with ministers if they ask for one. But it was the same stuff over and over and over again. When they realised that they weren’t making much progress with ministers, that it had been a collective decision that had been made and we were progressing with this policy, they went into senior official level.’ (Civil Servant, November 2010)  However, lobbying the current SNP government posed a new challenge for the industry. The party had never previously been in government and industry actors had not built the relationships with incoming ministers that they had with the previous Labour–Liberal Democrat coalition. In addition, key figures in the SNP government, including Justice Secretary Kenny MacAskill who initially led the push towards price-based interventions, appeared unwilling to afford the industry the same status in the policy process as under the previous administration. The establishment of the partnership agreement, and the broader framing of the policy debate under the Labour–Liberal Democrat coalition, had identified industry actors as key partners in the policy process. |
| Position of the industry in policy process |  |  |
| Success/ effectiveness of industry influencing activities. |  |  |

| Yoon, S. and T.-H. Lam (2012). "The alcohol industry lobby and Hong Kong's zero wine and beer tax policy." BMC Public Health 12(1): 717-717. | | |
| --- | --- | --- |
| **Category of Industry Activity** | **Summary of Data** | **Key quotations** |
| Policy approaches promoted (and arguments in favour) | Argued lower taxes would boost tourism and offer additional economic opportunities to Hong Kong.  Industry consistently highlighted the alleged positive health effects of wine.  Promotion of wine as health protective. | At the Legislative Council meetings in 2002, he repeatedly delivered the message that the high cost of quality wines and spirits in Hong Kong damages Hong Kong’s tourism sector. In order to convince politicians and government officials, the idea of Hong Kong becoming a regional hub in the alcohol trade was advanced. The coalition claimed that a lowered duty on wine would boost tourism and further strengthen Hong Kong’s image as a wine distribution centre.  “Drinking about two glasses of wine a day is beneficial to health and that is a major influence on the boom of the wine market in Hong Kong. . .Hong-Kong drinkers prefer red wine to white wine because of  more perceived health benefits associated with drinking red wine.”  On the policy front, the pro-business Liberal Party has been a key force behind the idea that drinking wine is healthy. At the heart of this move stood Party Chairman  James Tien Pei-Chun. Shortly after the alcohol tax reduction, he called for further tax cuts on wine. |
| Policy approaches opposed (and arguments against) | Opposed increases in/ restructuring of taxation on alcohol designed to meet Hong Kong’s fiscal shortfall. | The increase in alcohol taxation was met with immediate opposition from the alcohol industry. From the alcohol industry’s perspective, the tax represented a direct menace to their continued prosperity. The Hong Kong Beer Industry Coalition (HKBIC), which then comprised seven international brewers and importers, strongly opposed the tax increase on beer. In May 2001, the HKBIC claimed that “such an increase would adversely affect the livelihood of several hundred thousand people working in the beer, retail and catering industries”.  In its letter submitted to the Legislative Council in April 2002, the HKWIC argued that “wine is a staple consumer good enjoyed by a wide range of the population” and therefore such an increase “affects a large cross-section  of the population, not a small group of high-income earners.” They warned that the government would not receive the planned additional HK$ 70 million in revenue from the alcohol tax increase. Rather, they estimated that the tax increase would result in a loss of government revenue because there would be general signs of trading-down activities to cheaper priced alcohol |
| Industry self-definition/ positioning as a policy actor | Presented their industry as a key economic opportunity (as a wine distributor) and as a key driver of tourism. |  |
| Industry definition of other policy actors |  |  |
| Unity/cleavages across industry | Coalitions across industry and with other sectors (see below). |  |
| Influencing activities | Formed coalitions with other sectors.  Industry media strategy played a key role in their political strategy.  Used wide range of other influencing techniques. | In its efforts to lower (and eventually abolish) the tax  in question, the alcohol industry sought out industry allies such as the hospitality and trading industries and forged agreements with them to collectively advocate its position. Although forming alliances was not a new industry practice, it became an increasingly important strategy. Among others, the Hong Kong Wine & Spirits  Industry Coalition (HKWSIC) was notable for this. The coalition was first formed with the wine industry only and named as the Hong Kong Wine Industry Coalition (HKWIC) in 2002 “to lobby the government on industry  related issues such as alcohol duties.” Then the alcohol tax increase in 2001–2002 prompted a more typical formation of like-minded industry groups which shared similar vested interests. Fredric Dufour, the managing  director of Richemont Hong Kong, a transnational retailer of liquor products, tobacco and other luxury goods, became the first chairman of the HKWSIC.  Since late 2006, the industry elected to work closely with  local media in an attempt to garner the wide spectrum of  public support. For instance, daily newspaper titles such as  “80 percent tax on wine too high, says lawmaker” or “hospitality industry says it would pass on saving to long-suffering consumers” were indicative of how the coalition sought to garner and increase the level of public support.  In collaboration with business-friendly lawmakers, the alcohol industry employed a variety of instruments including reports, conferences, meetings, opinion pieces and letters to lucidly articulate the rationale for alcohol  tax elimination. |
| Channels of policy influencing activity | Identified key policy actors as channels of influence for industry policy initiatives and agendas.  Overseas governments. | In order to convince policy makers and political leaders,  the coalition needed a representative that could  provide legislative tax initiatives, help industry lobbyists gain access to lawmakers and senior government officials, demonstrate constituent support for alcohol tax reduction and testify on the industry’s behalf. Tommy  Cheung Yu-Yan, who holds the seat of the catering industry  functional constituency in the Legislative Council of Hong Kong, acted as a core policy link between the HKWSIC and the government, advocating for reductions in alcohol taxation.  Through meetings with Legislative Council members as well as officials in the Financial  Services and Treasury Bureau, the coalition provided a variety of supporting arguments and figures to substantiate their case.  In a separate move, the Australian and New Zealand Consulate- Generals in Hong Kong, the two major suppliers of wine to Hong Kong, sent protest letters to the government. They argued that “consumers will react to the tax increase by switching to lower taxed and untaxed beverages and to illicit sources of smuggled wine, defeating the aim of substantially raising revenue.” |
| Targets of policy influencing activity | Government, but also wider public opinion. | More importantly, in order to gain wider public support, they highlighted that the tax increase might significantly push up retail prices, which would eventually penalize the majority of ordinary beer drinkers. The HKBIC’s  attempt to rally the general public against the tax increase was well reflected in their public statement: “The HKBIC does not believe that it is fair to impose a duty rate increase, which affects a broad spectrum of ordinary consumers, while the duty rates on luxury products remain unchanged.” |
| Position of the industry in policy process |  |  |
| Success/ effectiveness of industry influencing activities. | Initially unsuccessful in reversing tax hike (up to mid 2000)  ….but later more successful with Hong Kong going from a relatively high tax to a zero tax environment in a very short space of time.  In part, this success was due to the absence of NGOs opposing the tax cuts (in contrast to their strong advocacy on other public health issues such as tobacco control). | Despite the alcohol industry’s vigorous lobbying, the Hong Kong government was initially resistant to the industry’s opposition primarily because alcohol tax was seen as a “stable source of government revenue”. […] For this reason, the tax rate on alcoholic beverages remained intact until the first half of the 2000s despite the industry’s political pressure and strong resistance.  By mid-2004, two factors appeared to have contributed  towards a new policy climate that favored a reduction in the alcohol tax. One was the robust economic rebound despite the severe economic fallout caused in part by the outbreak of Severe Acute Respiratory Syndrome (SARS)  in 2003. The Hong Kong economy exhibited a broad-based upturn in 2004 with a rise in real gross domestic product (GDP) of 8.7 percent amid a strong inflow of  capital funds and an upsurge in consumer spending. The other arguably more important factor was the persistent lobbying and political pressure from the alcohol industry. Over the years, the alcohol industry incessantly lobbied the government to lower the alcohol tax rate.  Faced with relentless industry lobbying, the Hong Kong Government then began to consider assessing the existing alcohol duty regime. In December 2004, the government launched a Public Consultation on the Duty on Alcoholic Beverages to seek feedback on the appropriate  level of alcohol taxation.  The corporative initiative appeared to have convinced  the government officials and senior policy makers. On 27 February 2008, Hong Kong entered a new era as it implemented a zero alcohol taxation policy. In his budget speech, newly appointed Financial Secretary John Tsang announced that he would scrap all duties on wine and beer. This move has made Hong Kong the only place in the world where wine and beer are completely untaxed.  Although the city’s changing financial circumstances and the Hong Kong Government’s strong propensity towards economic liberalism have in part contributed to such a dramatic transformation, the alcohol industry’s tactics and strategies were clearly the main drivers of the policy decision.  Whereas the alcohol industry had been constantly vociferous in lowering the alcohol tax, the second half of the 2000s witnessed striking changes in the political tactics and rhetoric that they employed to lobby for selective amendments to licensing laws and to the ways in which alcoholic products were taxed.  In view of the roles that local NGO actors actively played in the making of public health policy, it is surprising that they have apparently been totally absent in the policy debate on alcohol taxation. The lack of civil participation in creating policy dialogues and raising  concerns related to alcohol tax reduction lies in stark contrast to the vociferous mobilization and lobbies driven by the industry. |

| Hawkins, B. and C. Holden (2013). "Framing the alcohol policy debate: industry actors and the regulation of the UK beverage alcohol market." Critical Policy Studies 7(1): 53-71. | | |
| --- | --- | --- |
| **Category of Industry Activity** | **Summary of Data** | **Key quotations** |
| Policy approaches promoted (and arguments in favour) | Industry actors argue that the scale of the UK alcohol problem is overstated and restricted to a small minority of problem drinkers and policy responses should reflect this.  Alcohol problems are also linked to specific areas of the country which skews perceptions of the overall national picture.  Industry actors strongly advocate partnership based approaches versus legislation (see below). Where this couldn’t be avoided though extensive consultation was advocated.  Promoted tax (VAT/duty) as an alternative to MUP only if some sort of price-based intervention is unavoidable. (Some) industry actors shifted to this as the debate progressed.  Industry actors called for culture change around alcohol for example the acceptability of public drunkenness. Linked to individual responsibility and calls for more education and focussing on binge drinking v other harms.  Called for enforcement of existing laws (not more laws). E.g. under-age sales/ drink driving. | Virtually all the consultation responses framed the alcohol  problem in terms of a small minority of the population using alcohol excessively and  irresponsibly. The idea of a ‘sensible majority’ at the heart of the industry discourse has  obvious implications for the policy agenda. Framing the issue in this way leads logically to the conclusion that government policy ought to be targeted at that minority rather than at the entire population. .  The regional Health & Wellbeing Profiles 2008 provide graphic illustrations of the level of variation in the scale of problems associated with alcohol misuse in different parts of Scotland. 28% of all alcohol related hospital admissions in Scotland are in Glasgow whilst 9 out of 14 Health Boards have a lower than Scottish average level. Similarly, Glasgow accounts for 36% of all alcohol related deaths, whilst 11 out of 14 Health Boards are below the Scotland average.  “I think consultation is absolutely key to this. So let’s take alcohol disorder zones, something that the last government introduced. No-one has ever used it. What local authority would want to be called an alcohol disorder zone? *. . .* So, a daft piece of legislation; legislate in haste,  repent at leisure is clear. They didn’t consult properly; they’ve come out with something that nobody’s going to use. So the clear message, as far as I’m concerned, is: consult widely and listen to what people say in response to consultation. Because if you don’t, you can get yourself into an absolute muddle and we have.’ |
| Policy approaches opposed (and arguments against) | Industry actors proposed partnership approaches as explicit alternative to legislation.  They are explicitly opposed to MUP.  Legislation was argued to be ineffective and even counterproductive in undermining partnership.  Used comparisons with other countries (Nordics) to claim MUP would be ineffective.  Some opposed MUP (from an ideological position) as an unacceptable intervention in the free market. | ‘As a responsible company, Diageo believes that at a minimum industry and government should work together to root out all irresponsible promotions, whether in shops, supermarkets, clubs, bars or pubs. Going further, we believe that industry can play an important role in changing consumer attitudes to alcohol by working in partnership with government and civil society,  because partnership has the greatest chance of success in changing Scottish consumers’ relationship with alcohol. So we invite the Scottish Government to facilitate such a partnership, to ensure that alcohol can play the positive role in Scottish society that the Government says it desires.’  ‘You could argue that we should be in favor of a minimum unit pricing because potentially we’re going to make more money; but we fundamentally disagree with it because, you know, we think, on behalf of our customers, we should have the freedom to set our own prices. And that it shouldn’t be set such that it is totally fixed throughout the market in contrast to maybe  a floor price like duty and VAT. The reason we say that is if it’s minimum unit pricing, *. . .* what we think that does is it completely removes competition from the market, and therefore if you’re the largest retailer you’ve got a guaranteed market share three times bigger than us – that’s, you know, three times the sales of us at a price which we can’t do anything to vary to compete’ |
| Industry self-definition/ positioning as a policy actor | Industry actors highlight their positive (social and economic) contribution to UK society, especially as a key Scottish export product.  Claim to take pride in being responsible actors and to put policies in place to guarantee this. Irresponsibility is also bad for business.  Policies demonize alcohol (and the industry) but should demonize irresponsible use. | ‘There is a commercial interest to a lot of this, but I don’t believe it is all so cynical as to be wholly driven by commercial interest. I think that there is, on the part of some of the people involved, there is a genuinely held social responsibility view that this would be the right thing to do for society. [A representative of a large brewer] said in one of our council meetings not so long ago – we were talking about what we should be doing as an organization – and he said, ‘Well look, I don’t want my children growing up to be ashamed of what I do.’ And so I think he has got a genuine desire to reduce alcohol misuse.’ |
| Industry definition of other policy actors |  |  |
| Unity/cleavages across industry | No single unified position on policy issues (e.g. price). |  |
| Influencing activities | Framing the policy environment; individual responsibility; targeted interventions; partnership approaches; ineffectiveness of unfavoured policies. |  |
| Channels of policy influencing activity |  |  |
| Targets of policy influencing activity |  |  |
| Position of the industry in policy process | Historically, the industry is seen as a key partner in policy/ part of the solution to alcohol related harms, which are framed in industry friendly ways.  This framing shifted to health/ whole population approach in Scotland following activities of NGOs. This led in to an industry attempt to reassert their framing. | ‘That’s quite important because *. . .* if you look at the alcohol policy documents, certainly from the Westminster Government, but  even from the Scottish Government as recently as 2001, you’ll see that alcohol is identified as a problem, but the way that it’s framed in the policy documents is very much *. . .* in terms of the industry *. . .* . I think the industry is mentioned probably in the first sentence of the ministerial foreword and the frame of the problem is about most people drink alcohol responsibly and it’s really just this tiny minority that don’t, where we have to concentrate our efforts. Now, if that’s your frame then the policy solutions you come up with are going to be education and stuff like that. So a key advocacy task for us was to change the frame of the alcohol problem and to actually get politicians and the general public and the media thinking about [whole] population approaches to alcohol policy.’ |
| Success/ effectiveness of industry influencing activities. | Limited success in countering reframing of the policy debate, in Westminster, but not in Scotland. |  |

| Jiang, N. and P. Ling (2013). "Vested Interests in addiction research and policy. Alliance between tobacco and alcohol industries to shape public policy." Addiction 108(5): 852-864. | | |
| --- | --- | --- |
| **Category of Industry Activity** | **Summary of Data** | **Key quotations** |
| Policy approaches promoted (and arguments in favour) |  |  |
| Policy approaches opposed (and arguments against) | Tax increases, advertising restrictions and clean air legislation. | To oppose cigarette tax increases, which the public generally supports, 170 the tobacco industry broadened the cigarette tax issue to include alcoholic beverages and other consumer goods. The tobacco industry consistently identified the alcohol industry as a key partner and often the largest financial source for the anti-excise tax coalitions they created.  For tobacco advertising and promotion restrictions, the tobacco industry framed the restrictions as a violation of the First Amendment to make them relevant to the alcohol industry and advertising industries. The tobacco and alcohol industries were able to work in concert by affiliating with a powerful third party, the advertising and publishing industry, which took the lead in opposition to advertising restrictions. Although it is hard to conclude if the alcohol industry’s specific efforts in opposing tobacco advertising bans were associated with the failure of such bills in the Congress, the lobbying efforts around this legislation were elaborately planned. |
| Industry self-definition/ positioning as a policy actor |  |  |
| Industry definition of other policy actors |  |  |
| Unity/cleavages across industry |  |  |
| Influencing activities | Alliances with the tobacco industry. Used by transnational tobacco companies to support their policy agendas in area of perceived mutual interest. E.g. Partisan Project. | At these meetings, RJR emphasized that (1) tobacco  and alcohol industries share the same customers because “close to half of all people who are high volume buyers of distilled spirits are smokers”, (2) they share similar challenges in the political arena because public smoking restrictions affect alcohol customersand “today tobacco is the focus of prohibitionists. Alcohol follows close behind”, and (3) RJR’s Partisan Project could help distilled spirits industry members preserve their customers’ rights. RJR documents stated they “greatly enhanced the hospitality industry’s influence in 1989, evolving from leaders learning about issues to actively supporting smokers’ rights,” and “secured their [NLSA [National Liquor Store Association] and NLBA[National Licensed Beverage Association]] executive leadership’s endorsement of RJR’s Partisan smokers’ rights program.” |
| Channels of policy influencing activity | Astroturf organisations: e.g. Consumer Tax Alliance and Consumer Tax Forum.  Newsletters: ‘Choice’ & ‘Regulatory Watch’. | ‘The Consumer Tax Alliance is being formed to address the growing tax threat on consumer goods. CTA [Consumer Tax Alliance] is an alliance of business leaders and California consumers. The organization’s purpose is to gather data about the abuses and affects of excise taxes and provide this information to legislators, the news media, community and taxpayer organizations, and other opinion leaders.’  ‘Because this [alcohol] industry is so crucial to the success of the [CTF [Consumer Tax Forum] project, you may want to consider the active use of the Tobacco Institute and its members in soliciting participation. If there are tobacco industry contacts with corporate CEO’s and other senior level management in the alcoholic beverage group, they could be very helpful in generating support for the [CTF] project.’  Both newsletters included surveys on attitudes toward clean indoor air laws, tax increases on cigarettes and alcoholic beverages,  cigarette advertising bans, and the respondent’s propensity to take action. |
| Targets of policy influencing activity | Attempts to target policy makers and the wider public and to shape their attitudes. |  |
| Position of the industry in policy process |  |  |
| Success/ effectiveness of industry influencing activities. |  |  |

| McCambridge, J., et al. (2013). "Industry use of evidence to influence alcohol policy: a case study of submissions to the 2008 Scottish government consultation." PLoS Medicine / Public Library of Science 10(4): e1001431. | | |
| --- | --- | --- |
| **Category of Industry Activity** | **Summary of Data** | **Key quotations** |
| Policy approaches promoted (and arguments in favour) | Claim issues with alcohol are limited to small minority of problematic drinkers. | The WSTA claims to share the goal of the Scottish Government in reducing alcohol-related harm and seeks to work in partnership in developing an evidence-based approach to policy which can command high levels of  public support. It is necessary, they argue, ‘‘to tackle the minority with alcohol problems – the drinkers rather than the drink’’ and ‘‘policies which punish all drinkers for the misconduct of a few’’ would be unfair |
| Policy approaches opposed (and arguments against) | Whole population measures which affect the ‘moderate majority’ including MUP (see above). |  |
| Industry self-definition/ positioning as a policy actor | Partners to government as part of the solution (see above). |  |
| Industry definition of other policy actors |  |  |
| Unity/cleavages across industry | Widespread similarities industry actor submissions. |  |
| Influencing activities | Industry actors claim to support evidence based policy but misrepresent strong evidence for unflavoured policies and question evidence used by government to support. This is not on the basis of a review of the evidence though.  Promote weak evidence in support of their position, often conducted by themselves.  Unsubstantiated claims made about adverse effects of policy proposals.  Promote alternative policy measures to MUP which lack supporting evidence (principally targeted harm reduction measures). | These statements imply some evaluation of evidence, which is not presented in any of the submissions. Only in one instance was this omission declared: ‘‘We have not  sought to carry out a detailed analysis of the evidence base referred to in the  strategy in our response but we would observe that it is very selective’’  According to the PG, ‘‘There is a raft of contradictory evidence of the influence of price and promotions on harm. In the absence of strong evidence, it seems imprudent to tackle alcohol misuse by acting against price and promotions’’ [paragraph 4.13].  Similarly, the large literature demonstrating the limited impact of education […] is misrepresented. According to the WSTA, ‘‘Many commentators have attacked education as being ineffective in changing drinking behaviour. In fact there has been very little research in the area’’ [page 32].  Their emphasis on public support makes opinion polls a key form of evidence. The SBPA [Scottish Beer and Pub Association], Morrisons, and ASDA commissioned opinion polls themselves. ASDA presents full results of a survey of 10,109 customers in the form of a tabulation of responses to four questions [page 3].  Market research is also presented as intervention evaluation evidence. The  outcome data in these reports are by their nature unable to provide evidence of changes in behaviour or reducing harms, as is implied. For example, referring to one of their campaigns, Diageo suggest ‘‘Evaluation has shown the advertising to have a positive impact: more than 60% of those surveyed by the media evaluation agency Millward Brown said they were more likely to consider drinking responsibly following the adverts’’ [page 9].  Portman Group: ‘‘Adopting a population-wide approach may not only fail to reduce misuse but it could perversely contribute to an increase in unhealthy drinking patterns and unregulated trading with the associated criminal activities [paragraph 4.8] … [increasing off-licence purchase age] could foster a feeling of resentment among young adults. It could also increase the appeal of alcohol to young people by creating a ‘mystique’ surrounding alcohol. Turning alcohol into a ‘forbidden fruit’ will only enhance its appeal to young adults looking to find ways of escaping the problems in their lives’’ [paragraph 5.28].  Diageo: We strongly believe that a system of co-regulation is the most appropriate and effective approach to tackling alcohol misuse. Under co-regulation, the Government and the alcohol industry draw up standards together, which are strictly monitored and enforced, both within the industry and by Government through existing laws and regulations. But we also believe that individuals must take responsibility for their own actions, a principle which is unfortunately largely lacking in the discussion paper’’ [page 3].  SAB-Miller: ‘‘There are less intrusive means by which the Scottish government can achieve its objectives than by imposing broad, population-based policies.  These less intrusive means include education efforts on the laws against underage drinking, disorderly conduct, and sales to intoxicated or underage people, coupled with consistent and rigorous enforcement of these existing laws. Education and enforcement should be the cornerstone of Scotland’s strategy to tackle alcohol misuse’’ [paragraphs 11 and 12]. |
| Channels of policy influencing activity | Trade associations (WSTA) SAPROS (PG) and individual companies via consultation responses. |  |
| Targets of policy influencing activity | Government consultation in raising MUP. |  |
| Position of the industry in policy process | Partners to government in the policy process; part of the solution not part of the problem. |  |
| Success/ effectiveness of industry influencing activities. | Limited in the case given the decision by the Scottish government to progress MUP. However, the same arguments were used and the UK government explicitly cited lack of evidence in its decision not to proceed with MUP. |  |

| Hawkins, B. and C. Holden (2014). "'Water dripping on stone'? Industry lobbying and UK alcohol policy." Policy and politics 42(1): 55-7 | | |
| --- | --- | --- |
| **Category of Industry Activity** | **Summary of Data** | **Key Quotations** |
| Policy approaches promoted (and arguments in favour) |  |  |
| Policy approaches opposed (and arguments against) |  |  |
| Industry self-definition/ positioning as a policy actor | Key partners to government in the policy making and implementation processes. |  |
| Industry definition of other policy actors |  |  |
| Unity/cleavages across industry | Perceived benefit of acting collectively where possible. |  |
| Influencing activities | Seek to build long-term relationships with key decision makers.  Formal events such as government consultations, private meetings, party conference events and APG [All Party Group] meetings.  More informal but frequent/ regular meetings with policy makers.  Personal contacts and networks between industry actors and policy makers are important.  The provision of ‘policy goods’ (e.g. information expertise, and policy delivery via Drinkaware and Portman Group). | Contacts were established between industry actors, MPs, officials and ministers through what were described by respondents as ‘standard lobbying practices’. By this they meant a range of activities, including both written submissions and face-to-face meetings of various types. Written submissions or briefing documents prepared by companies or trade associations often served as a prelude to a meeting. […] In addition, industry actors attended lunches or breakfasts with ministers, MPs and/or civil servants and invited politicians to visit company sites.  The sustained contact between the industry and government establishes the norm that industry actors should be part of the policy process. This is evident in the  widespread acceptance among politicians, and even some NGO respondents, that  industry actors are key partners in delivering policy solutions. |
| Channels of policy influencing activity | Via trade associations but large companies may act unilaterally.  Both bilateral and multi-lateral meetings take place.  Outside agencies and consultancies are also used.  SAPROs. |  |
| Targets of policy influencing activity | All stages of the policy process and all relevant policy actors, including opposition parties.  Different approaches taken to different actors such as civil servants and ministers.  Contact with officials is more frequent than with ministers. | Alcohol industry actors seek to involve themselves at every stage of the policy process  from initial consultations, through the deliberative and legislative phases to the final  implementation of new measures.  Industry actors seek access  to the entire range of political actors involved in policy making: civil servants, MPs,  regional and local government actors, special advisers and ministers, including those  at the very highest level of government. |
| Position of the industry in policy process | Privileged actors in the policy process over e.g. NGOs. Particularly large companies who enjoyed greater/ more frequent access.  Seen as stakeholders who must be at least consulted on policy development and accommodated where possible. | Representatives from some of the largest and most  prominent producer and retailer organisations occupy a particularly privileged position,  being invited to so-called ‘pre-consultation discussions.’  This [regular meetings with policy makers] was seen by respondents as a normal and perfectly legitimate part of  the policy process. It reflects the status afforded to industry actors as key stakeholders in the policy process. Public health activists claimed that they are simply not able to obtain the level of access to government granted to industry actors.  It is common practice for civil servants and government ministers to seek engagement with industry in an attempt to gain industry ‘buy-in’.  The fact that industry actors are afforded a seat at the table does not necessarily mean  that their views are taken on board or that they are effective in shaping policy. On  certain issues, policy will run counter to their interests. Nevertheless, there appears to  be an acceptance on the part of government that they should at least attempt to work in collaboration with industry, and to get industry to buy in to a policy wherever possible. |
| Success/ effectiveness of industry influencing activities. |  |  |

| Hawkins, B. and J. McCambridge (2014). "Industry actors, think tanks, and alcohol policy in the United kingdom." American Journal of Public Health 104(8): 1363-1369. | | |
| --- | --- | --- |
| **Category of Industry Activity** | **Summary of Data** | **Key quotations** |
| Policy approaches promoted (and arguments in favour) | Parenting styles and individual responsibility (e.g. ‘Why should responsible drinkers pay more’ campaign). |  |
| Policy approaches opposed (and arguments against) | Oppose MUP and create false dichotomy with other (ineffective approaches) such as parenting styles. | Like industry actors elsewhere, the report judges that the evidence on MUP is “not conclusive.” |
| Industry self-definition/ positioning as a policy actor | Legitimate producers of science and stakeholders in the policy process. |  |
| Industry definition of other policy actors | Ignored mainstream peer reviewed research. |  |
| Unity/cleavages across industry | Evidence of collaboration across the producer sector (via Drinkaware/ WSTA) and the retail sector (via Addaction and trade associations (ACS)/ WSTA) but this is largely circumstantial. |  |
| Influencing activities | Funding the production of policy relevant evidence at key junctures in the policy making process (i.e. the development of the UK Government Alcohol strategy & implementation of MUP).  Mimicking the legitimate scientific literature (e.g. using same title as a British Medical Association funded study ‘Under the Influence’); but with methodological weaknesses.  Selective use of legitimate reviews (e.g. excluding seminal WHO reviews).  Publicity/ media material released ahead of reports to prevent scrutiny and rebuttal.  Overall effect is to create a separate, circumscribed & self-referential literature produced by industry funded/ connected bodies which ignores the (unfavourable) international research consensus. | The literature review omitted  the World Health Organization sponsored peer-reviewed summary of the alcohol policy evidence base and accessed instead industry-funded publications, including a 2009 Centre for Economics and Business Research report on MUP commissioned by SABMiller, alongside other outputs by the Wine and Spirit Trade Association and 2 alcohol industry social aspects and public relations organizations: the International Centre for Alcohol Policies and Drinkaware.  Notwithstanding the  limitations of these reports, their relevance to ongoing policy debates was explicitly and forcefully articulated in ways designed to influence thinking about, and decision-making on, MUP. They were launched at the heart of  government and promoted at the conferences of the 3 largest United Kingdom---wide political parties. |
| Channels of policy influencing activity | Think Tanks: Demos, London Economics CEBR; (Adam Smith Institute, Institute of Economic Affairs cited in background/ previous studies).  SAPRO’s: Drinkaware.  Multiple reports and outputs funded by SABMiller published in quick succession.  Reports facilitated by industry funded 3^rd^ sector bodies such as ACS. |  |
| Targets of policy influencing activity | Parliamentarians and policy makers; the wider policy community and societal discourses on alcohol harm and individual responsibility via the media.  Launch events in Parliament.  Party conference fringe events. |  |
| Position of the industry in policy process |  |  |
| Success/ effectiveness of industry influencing activities. | MUP dropped with an explicit and highly contestable reference to the lack of supporting evidence for MUP, qualified by key industry argument, as the rationale for not proceeding. | The formal announcement of  the decision to halt plans for MUP implementation accessed a lack of evidence that MUP would achieve desired reductions in alcohol harms and spurious alcohol industry  concerns about the effects of MUP on moderate consumers:  We do not yet have enough concrete evidence that its introduction would be effective in reducing harms associated with problem drinking—this is a crucial  point—without penalising people who drink responsibly. |

| Katikireddi, S. V., et al. (2014). "Changing policy framing as a deliberate strategy for public health advocacy: a qualitative policy case study of minimum unit pricing of alcohol." Milbank Quarterly 92(2): 250-283. | | |
| --- | --- | --- |
| **Category of Industry Activity** | **Summary of Data** | **Key quotations** |
| Policy approaches promoted (and arguments in favour) | Focussed on individual level consumption and responsibility, and fostering moderate drinking.  Government action should be targeted and not punish those behaving (apparently) responsibly.  Underlying goal to achieve reduced harm was cultural change around UK society’s relationship to alcohol.  The hybrid discourses accepted the anti-MUP contentions about the goal of policy, i.e. to foster responsible drinking (e.g. position of Molson-Coors). | ‘Our aim is to ensure that moderate consumption continues to be part of normal healthy life in Scotland, and that misuse is regarded as unacceptable behaviour.’  These critics suggested alternatives that would not affect overall levels of population consumption but instead would target those  who “misused” alcohol, with price measures such as a ban on below-cost sales (which were presented as interfering less in the alcohol market’s functioning).’  They also frequently recommended non-price alternatives, with education-based measures particularly  prominent. |
| Policy approaches opposed (and arguments against) | MUP was only opposed by industry actors (some of whom supported it).  They questioned the extent of alcohol related harm and the need for public policy responses.  They focused on a narrower range of harms (harmful, binge and youth drinking) limited to specific sub-populations.  The hybrid discourses (of industry actors supporting MUP) accepted the anti-MUP contentions about the current problem posed by alcohol (i.e. underplaying that scale of the problem). | Overarching competing framings were apparent and were used by policy stakeholders to help support their position on minimum unit pricing: a framing that presented minimum unit pricing in a positive manner and a critical framing that was used only by industry actors (Figures 1 and 2). In addition, a third hybrid framing was adopted by industry actors who supported minimum unit pricing but retained most aspects of the critical frame (Figure 3). |
| Industry self-definition/ positioning as a policy actor |  |  |
| Industry definition of other policy actors |  |  |
| Unity/cleavages across industry | Some companies and trade associations are hard line opponents of MUP but others were formally in favour of it, but shared much of the ‘hard line’ discourse on the problem definition and the policy solution. |  |
| Influencing activities | A shift in framing was key to moving the policy debate along. Thus, counter framing by industry was also vital.  Questionable interpretation of relevant science (e.g. epidemiological data) which differed from that of the public health actors. | Changing the framing of the policy debate did not appear to be straightforward and required those advocating public health action to continually challenge the industry framing. Many advocates saw the importance of securing a shift in framing as an important victory, especially the articulation of a population health framing in official Scottish government policy documents.  Our research also showed the diverse and contradictory ways that research evidence can be presented within a policy debate. The availability  of different sources of epidemiological data and the choice of different time periods over which to illustrate trends helped policy actors present the policy issue in markedly different ways—as either a crisis requiring urgent action or a problem that was being resolved. |
| Channels of policy influencing activity | Companies themselves, trade associations and SAPROs. |  |
| Targets of policy influencing activity | Policy makers and the wider public. |  |
| Position of the industry in policy process |  |  |
| Success/ effectiveness of industry influencing activities. |  |  |

| Kypri, K., et al. (2014). "Public, official, and industry submissions on a Bill to increase the alcohol minimum purchasing age: A critical analysis." International Journal of Drug Policy 25(4): 709-716. | | |
| --- | --- | --- |
| **Category of Industry Activity** | **Summary of Data** | **Key quotations** |
| Policy approaches promoted (and arguments in favour) | Asserted an individualised framing of alcohol related harm. | Diageo, one of the largest alcohol companies in the world, primarily involved in the production, importation and marketing of spirits and spirit-based mixed drinks, wrote that “the harm from alcohol arises from the persons who abuse it and not from the product itself”. This was a common refrain in submissions from the alcohol industry. |
| Policy approaches opposed (and arguments against) | The industry opposed increase in minimum purchase age.  Industry actors made frequent, unsupported claims that this would be ineffective. Where evidence was cited it often came from industry sources.  Other arguments focussed on adult rights being infringed and that pubs represented safe/controlled environments in which to drink. | Brewer Lion Nathan: “There is no research which has yet established a link between a binge drinking culture and any legislated minimum age of purchase” (p.6), and cited an Alcohol Advisory Council document in support of this claim.  [Across all types of actors, not just industry] There were 14 types of argument used to argue against the proposed increase in the MPA [minimum purchasing age], several of which were empirical claims (e.g., it would not reduce drinking among young people) while others were normative claims (e.g., it is up to individuals to control their drinking). In |
| Industry self-definition/ positioning as a policy actor |  |  |
| Industry definition of other policy actors |  |  |
| Unity/cleavages across industry |  |  |
| Influencing activities |  |  |
| Channels of policy influencing activity | Individual companies and trade association, via multiple submissions. | There were 12 similar submissions from the Hospitality Association of New Zealand (HANZ), including one from head office and11 from its 21 local branches. This approach appears to reflect a strategy of maximising the number of submissions representing the interest group with probably the most to lose if the MPA were increased. All of the submissions expressed opposition to increasing the MPA.  The alcohol industry alone produced over 40% of all submissions opposing the Bill. Many of those were from regional offices of the same bodies, and none gave a balanced account of the research evidence. Legislators must ensure they are not fooled by such ploys. |
| Targets of policy influencing activity | Government consultation on raising MPA. |  |
| Position of the industry in policy process |  |  |
| Success/ effectiveness of industry influencing activities. | Industry faced only weak and fragmented oppositions to its positions. | Local government, an important stakeholder in the context of a country without state or provincial jurisdictions, was practically silent on the issue. In contrast to the fragmented and inconsistent response from government and NGOs, the alcohol industry was organised and united, with multiple submissions from the sector with most at stake, namely the hospitality industry, and supporting submissions from the manufacturing, import, and wholesale sectors. |

| Sornpaisarn, B. and C. Kaewmungkun (2014). "Politics of alcohol taxation system in Thailand: behaviours of three major alcohol companies from 1992 to 2012." The International Journal of Alcohol and Drug Research 3(3): 210-218. | | |
| --- | --- | --- |
| **Category of Industry Activity** | **Summary of Data** | **Key quotations** |
| Policy approaches promoted (and arguments in favour) |  |  |
| Policy approaches opposed (and arguments against) | The current tax regime on alcohol products, which had a differential effect on different product types and led to product shifting to lower taxed (thus cheaper products).  Alcohol and Health NGOs favoured maintaining the existing regime. |  |
| Industry self-definition/ positioning as a policy actor |  |  |
| Industry definition of other policy actors |  |  |
| Unity/cleavages across industry | Highly concentrated oligopolistic market controlled by 3 companies (92% market share); disagreements between companies on tax issue due to different product portfolios. Existing regime favoured one company versus the other two. | The three major alcohol companies were not in agreement in their positions, because the proposed alcohol taxation methods provided different competitive advantages for different companies.  The major alcohol companies that were disadvantaged by the Thai differential tax rate policies tried to challenge the current method of taxation. Had they been successful, the tax rates levied on their products might have been lowered or made equal to those of their competitors, which would have improved their competitive advantage. Conversely, Company A, which benefited from the current method of taxation, never challenged any government decisions regarding alcohol taxation. This behavior indicates that alcohol companies advocate for taxation policies that favor the positions of their products. |
| Influencing activities | Over production prior to tax increases to avoid the effects of the policy and to de facto delay tax increases.  Direct access to the Thai Prime Minister.  Use of overseas researchers to provide policy relevant evidence favourable to the industry.  Contact with key legislators. | The largest import company, which is in Group C, obtained access to the Thai Prime Minister in early 2005 after it donated a large amount of money to help the government with tsunami relief. The company’s Australia-based researchers provided information which argued for a change in the current alcohol taxation system in order to protect consumers’ health. The proposed tax method would result in lower taxation of the company’s products, but this fact was not mentioned. The Prime Minister intended to publicly support this proposal, but his cabinet dissolved before the tax system was changed.  Company B’s alliance with a member of the National Legislative Council influenced the parliamentary process by which the Excise Taxation Bill, which proposed a change to the current alcohol taxation method, was considered. |
| Channels of policy influencing activity | Overseas researchers.  Key policy actors including the Thai Prime Minister | With respect to Case 1, the largest import company, which is in Group C, obtained access to the Thai Prime Minister in early 2005 after it donated a large amount of money to help the government with tsunami relief. The company’s Australia-based researchers provided information which argued for a change in the current alcohol taxation system in order to protect consumers’ health. The proposed tax method would result in lower taxation of the company’s products, but this fact was not mentioned. The Prime Minister intended to publicly support this proposal, but his cabinet dissolved before the tax system was changed. |
| Targets of policy influencing activity | Thai PM and Key legislators (see above). |  |
| Position of the industry in policy process |  |  |
| Success/ effectiveness of industry influencing activities. | Some evidence of successful industry influencing activities in the legislative process. | Two unusual events occurred. First, a member of Parliament called seven meetings of the parliamentary commission considering this bill over a four-week period. Although he failed to advocate successfully for changing the tax method, which would haves provided the most benefit for Company B, he was more successful in his support of the second-most favorable option: the equal tax rate. A proposal for this change was registered for official consideration in Parliament for the final step towards becoming national legislation; however, in the second unusual event, the proposal was removed from the parliamentary agenda on the last day the term (i.e., December 21, 2007), resulting in the dismissal of the entire excise bill. No explanation was provided for the removal of the bill. |

| Savell, E., et al. (2016). "How does the alcohol industry attempt to influence marketing regulations? A systematic review." Addiction 111(1): 18-32. | | |
| --- | --- | --- |
| **Category of Industry Activity** | **Summary of Data** | **Key quotations** |
| Policy approaches promoted (and arguments in favour) | Industry actors advocated self-regulatory regimes and education via SAPROS such as PG and Drinkaware….as part of an individual framing of the problem (see quotes below). These, it is argued, negate the need for formal regulation.  Question the evidence for restrictions on marketing.  Argued that existing regulations are adequate and more/ new laws are not needed, just better enforcement of the current regimes.  Advocated partnership based approach.  Interventions to be targeted against the small number of problematic drinkers.  See Table 3. | The promotion of self-regulatory measures is designed to reduce political pressure for and pre-empt formal regulation and was identified in numerous jurisdictions. For example, we found evidence of voluntary codes being developed and promoted by individual companies and by industry groups in the UK, Ireland, the Netherlands and  transnationally.  Another technique involves the promotion of non-regulatory initiatives such as education programmes delivered through standalone  websites (for example, SABMiller’s [www.Talking](http://www.Talking)Alcohol.com) or more developed corporate social responsibility (CSR) initiatives. For example, Diageo’s Responsible Drinking Fund, which in 2009 claimed to have led or supported more than 130 prevention programmes in more than 40 countries, covering ‘education, public awareness, and responsible retail practices’.  The focus on a small number  of alcohol misusers provides the AI with a frame that has the potential to invalidate the current focus of health policy; the AI argues that population-level approaches, such as taxation or restrictions on advertising, penalizes moderate drinkers because of a ‘few people’ who consume alcohol in an irresponsible way and that these approaches do not tackle alcohol misuse effectively. |
| Policy approaches opposed (and arguments against) | Marketing regulation; advertising restrictions; whole population measures.  Argues that regulations may have unintended negative consequences and lack evidence.  20 arguments under 5 main frames used to influence marketing regulation; see Table 3. | The focus on a small number of alcohol misusers provides the AI with a frame that has the potential to invalidate the current focus of health policy; the AI argues that population-level approaches, such as taxation or restrictions on advertising, penalizes moderate drinkers because of a ‘few people’ who consume alcohol in an irresponsible way and that these approaches do not tackle alcohol misuse effectively.  This review identified 20 separate arguments grouped into five main frames (Table 3): ‘regulatory redundancy’ (asserting that proposed policies are unnecessary), ‘legal’ (questioning the legality of policies (the implicit cost for government), ‘negative unintended consequences’ (direct and indirect compliance costs associated with proposed policies), ‘complex policy area’ (policies, and the issues surrounding them, are presented as highly complicated) and ‘insufficient evidence’ (questioning the strength of evidence supporting policies). |
| Industry self-definition/ positioning as a policy actor | Differentiation of alcohol from tobacco.  Argument made that the industry, its marketing and alcohol itself has an overall positive effect on society.  See Table 3. | ‘Alcohol is not like cigarettes; it is capable of being misused but when drunk in moderation it is perfectly compatible with a healthy lifestyle’. This argument has also been used regarding minimum pricing, dealing with alcohol misuse and raising the legal drinking age. |
| Industry definition of other policy actors |  |  |
| Unity/cleavages across industry |  |  |
| Influencing activities | Twenty different tactics identified across five major strategies used to influence marketing regulation. See Table 2.  Similarities and differences with the tobacco industry  CSR activities are crucial to industry argument and influencing activities | This review identified 20 separate tactics falling under five main strategies (Table 2), which we have termed as follows: ‘information’ (providing or misrepresenting evidence), ‘constituency building’ (forming alliances with other sectors, organizations, or the public to give the impression of larger support for the industry’s position), ‘policy substitution, development and implementation’ (proposing, supporting or helping to implement alternative policies), ‘legal’ (using  the legal system) and ‘financial incentive or disincentive’ (offering direct or indirect monetary incentives or threatening financial withdrawal).  A variety of information strategies were used across multiple jurisdictions. These include direct and indirect lobbying of policymakers and establishing  collaborative working arrangements with policymakers, and a variety of efforts aimed at shaping and manipulating the evidence base. The latter included commissioning, writing or disseminating research/publications or more technical reports, the selective citation and omission of evidence, contesting the evidence used to support policy and the efforts to remove ‘troubling’ phrases such as ‘alcohol and other drugs’ from the official lexicon. Using or raising the prospect of legal action against a proposed regulation was documented only in Thailand and France, but there is also evidence of the AI [alcohol industry] attempting to shape international trade and investment agreements (specifically the General Agreement on Trade in Services (GATS) with a view to reducing restrictions on alcohol distribution and advertising).  We identified 13 common tactics used by both the AI and TI when attempting to influence marketing regulation, in addition to five tactics used only by the TI and seven unique to the AI. Similarly we also identified 13 common arguments used by both industries, along with four arguments unique to the TI, and seven (three of which formed the new frame ‘complex policy area’) which had been used only by the AI.  The review also found that many arguments were supported by CSR activities. CSR tends to be used strategically by an industry to prevent the introduction of legislation. By acting as vehicles for the promotion of arguments, CSR activities such as self-regulatory codes work politically as agenda-setting devices which frame issues and shape policy debates. The AI’s emphasis on CSR highlights its value in maintaining industry credibility and forming relationships (CSR partnerships are likely to create further opportunities for cooperation) ahead of regulation. |
| Channels of policy influencing activity | Industry actors, trade associations and SAPROs. |  |
| Targets of policy influencing activity | Government. |  |
| Position of the industry in policy process | Partners in the policy process, co-regulators with government. |  |
| Success/ effectiveness of industry influencing activities. |  |  |

| Avery, M. R., et al. (2016). "Mechanisms of influence: Alcohol industry submissions to the inquiry into fetal alcohol spectrum disorders." Drug & Alcohol Review 35(6): 665-672. | | |
| --- | --- | --- |
| **Category of Industry Activity** | **Summary of Data** | **Key quotations** |
| Policy approaches promoted (and arguments in favour) | Industry self-regulation and initiatives such as voluntary prevention programmes, e.g. Drinkwise labelling initiatives. Criticised as public relations and ineffective by the authors.  Downplaying the scale of the problem and thus the need for government action to counter this.  Sought to highlight already high levels of awareness of harms of drinking in pregnancy amongst pregnant women and resulting behaviour change (obviating the need for labels). Figures questioned by authors. | ‘As part of a DrinkWise Australia strategy, many sectors of the alcohol industry are voluntarily placing alcohol and pregnancy information labels on products preferred by female consumers. The DrinkWise Labelling initiative includes written and visual images as well as direction to a relevant website where additional detailed information is available’ (AHA (WA), page 11).  ‘Awareness of the potential negative impact of alcohol in  pregnancy is already very high, amongst women at least. This is shown by the great majority of pregnant women (97.5%) who already alter their alcohol intake positively, either totally abstaining (52%) or reducing the amount consumed (45.5%) according to the 2010 National Drug Strategy Household Survey’ (DSICA, page 2 and 3). |
| Policy approaches opposed (and arguments against) | Mandatory labelling regimes; which may have potentially adverse side effects (3/5 submissions by industry bodies).  Question effectiveness of any warning labels (2/5 industry submissions ). | Three of the alcohol  industry submissions (AWRI [Australian Wine Research Institute]; WFA [Winemakers’ Federation of Australia]; AHA (WA) [Australian Hotels Association of Western Australia] suggested that alcohol warning labels could have adverse effects for pregnant women, including prompting the unnecessary  termination of pregnancies, and the efficacy of alcohol warning labels is questioned across all of the submissions.  In one instance, the WFA used the higher prevalence of FASD [fetal alcohol spectrum disorders] in Aboriginal and Torres Strait Islander communities to question the need for population-wide exposure to mandatory warning labels, ‘We [WFA] therefore question why the entire population is subjected to mandatory labels on their products when they are shown to drink responsibly, with few negative consequences’ (WFA, page 17).  ‘Research suggests that telling an individual that a  behaviour is harmful or providing information about the risk associated with a behaviour is insufficient to affect an individual’s actions, while increasing an individual’s knowledge about a health risk does not necessarily cause  that individual to change or modify negative or risky behaviour [Engs 1989]’ (AWRI, page 18). |
| Industry self-definition/ positioning as a policy actor |  |  |
| Industry definition of other policy actors | Attempts to undermine public health researchers and organisations, especially National Alliance for Action on  Alcohol (NAAA) and the National Drug and Research Institute and some individual researchers. | ‘There is a worrying trend for what can only be described as  an anti-alcohol lobby to portray all alcohol as bad all the time and to dismiss as industry spin the legitimate social and (proven) health benefits of moderate consumption. There is a credible risk that unwarranted and over-dramatic label warnings will become a part of that campaign’ (Brewers, page 18, submission part two). |
| Unity/cleavages across industry | Differences in focus/ emphasis as opposed to clear differences in positions.  Authors find industry to be united, in keeping with previous literature on corporate strategy. | The current findings reflect established patterns of: (i) inter-agency collaboration to present united messages and  downplaying the role of alcohol in associated harms  (e.g. DrinkWise membership) |
| Influencing activities | Submissions to Australian Government House of Representatives Standing Committee of Social and Legal Affairs commenced an Inquiry into the prevention, diagnosis and management of FASDs. | The current findings reflect established patterns of: (i) inter-agency collaboration to present united messages and downplaying the role of alcohol in associated harms (e.g. DrinkWise membership); (ii) establishing and utilising SAPROs to project corporate social responsibility (i.e. DrinkWise); (iii) attacking the credibility of independent researchers; and (iv) endorsing the use of benign interventions, such as the current voluntary warning label system, which are known to be ineffective and have negligible impact to profit. |
| Channels of policy influencing activity | Submissions from 5 industry trade associations:  (i) The Winemakers’ Federation of Australia (WFA); (ii) the Brewers Association of Australia and New Zealand Inc. (Brewers); (iii) the Distilled Spirits Industry Council of Australia (DSICA); (iv) the Australian Wine Research Institute (AWRI); and (v) Australian Hotels Association of Western Australia (AHA-WA).  Use of SAPROs. | (ii) establishing and  utilising SAPROs to project corporate social responsibility  (i.e. DrinkWise). |
| Targets of policy influencing activity | Government via committee. |  |
| Position of the industry in policy process |  |  |
| Success/ effectiveness of industry influencing activities. | The post-consultation report advocated additional measures unfavoured by industry (mandatory inclusion of improved warning labels on products) but this didn’t translate into government policy, which continued with the existing industry friendly approach.  Voluntary labelling regime maintained. | Despite the recommendation to include warning labels on all alcohol products, in June 2014 Australian governments and the New Zealand Government, through the Legislative and Governance Forum on Food Regulation, recommended that the existing voluntary measures by industry be allowed to continue and be reviewed in two years. The Government did announce a National FASD Action Plan. However this Plan did not include preventive measures, such as warning labels or other alcohol controls.  In 2011 the Australian government implemented  a voluntary labelling trial giving the alcohol industry two years to voluntarily implement pregnancy warning labels on all alcohol product labels. The two year deadline passed with variable uptake and little indication of mandatory labelling in future. In fact, the most recent audit of Australian alcohol warning labels indicates that they are used on only one in three products. Moreover, in July 2014 the Australian Government announced that they would extend the voluntary labelling regime by another two years. |

| Zatoński, M., et al. (2016). "Framing the policy debate over spirits excise tax in Poland." Health Promotion International. | | |
| --- | --- | --- |
| **Category of Industry Activity** | **Summary of Data** | **Key quotations** |
| Policy approaches promoted (and arguments in favour) |  |  |
| Policy approaches opposed (and arguments against) | Increase of 15% in duty on spirits introduced by the Polish government.  Two overarching frames in relation to the policy: health and economic, with most statements falling under the economic frame. Third, symbolic frame (see article) was much less commonly evident in articles examined. (See Tables for more specific arguments).  Industry sought to focus arguments on the economic frame. | Main industry arguments deployed (number of mentions) from Table 1:  Legislation increases black market  Divisions in alcohol industry  Highlight positive effects of alcohol on society  Highlight tax contribution of industry  Alcohol prices already higher than elsewhere  Minimise perceptions of scale of alcohol problem  Provide employment  The TACs successfully promoted the economic frame, which gave them, as economic actors, an advantage in setting the terms of the debate. The industry selectively cited powerful precedents, echoing the  arguments used during the excise tax debate in Finland  in 2003, when the industry successfully lobbied for a reduction in taxes because of the threat of imports of  cheaper products from Estonia, soon to join the  European Union.  The flurry of activity by industry representatives maintained a high profile in the media where they were portrayed as sources of expertise and informed opinion, and stood in stark contrast to the lack of public health voices. |
| Industry self-definition/ positioning as a policy actor | Key economic and political actors with a positive impact on society. |  |
| Industry definition of other policy actors |  |  |
| Unity/cleavages across industry | Clear unified industry position (see below). |  |
| Influencing activities | Efforts to frame policy debates in industry favourable terms via the media. | The alcohol industry imposed economic considerations as the principal frame of the excise tax policy debate in Poland, making it easier to foreground the negative potential outcomes of a tax increase, and successfully placing itself as the source of information most frequently echoed by the media. |
| Channels of policy influencing activity | Key trade associations. | The Polish Spirits Industry (ZPPPS) is the largest industry trade association representing over 70% of the market of spirits in Poland. The Polish Vodka Association (PVA) is an organisation devoted to the global promotion of Polish vodka. |
| Targets of policy influencing activity | Politicians and the wider public whose support they require for policies. |  |
| Position of the industry in policy process |  |  |
| Success/ effectiveness of industry influencing activities. | Clear unified industry message was successful against the equivocation of government. | Meanwhile, the government’s counterarguments were less clear and relied on complex calculations that were difficult to interpret and suggested only minimal decreases in consumption and increases in revenue. Faced with this, the media picked up the much clearer suggestions from industry officials, so that industry sources, mostly from trade associations, became the most frequently quoted group of stakeholders in the print media.  The findings suggest that the public debate on alcohol policy in Poland was dominated by the views of the alcohol industry and other opponents of the legislation. The legislation eventually passed and the increase was implemented. However, the government was forced to promise that the spirits excise tax will not be increased again  in the next two years (Author Unknown, 2014). |
